# Characterizing the population structure and genetic diversity of maize breeding germplasm in Southwest China using genome-wide SNP markers

**DOI:** 10.1186/s12864-016-3041-3

**Published:** 2016-08-31

**Authors:** Xiao Zhang, Hua Zhang, Lujiang Li, Hai Lan, Zhiyong Ren, Dan Liu, Ling Wu, Hailan Liu, Jennifer Jaqueth, Bailin Li, Guangtang Pan, Shibin Gao

**Affiliations:** 1 Maize Research Institute, Sichuan Agricultural University, Chengdu, 611130 Sichuan People’s Republic of China; 2 Key laboratory of Biology and Genetic Improvement of Maize in Southwest Region, Ministry of Agriculture, Chengdu, 611130 Sichuan People’s Republic of China; 3 DuPont Crop Genetics Research, Route 141 and Henry Clay Road, Wilmington, DE 19880-0353 USA; 4 Mianyang Academy of Agricultural Sciences, Mianyang, 621023 Sichuan China

**Keywords:** Maize, Southwest China, SNP array, Population structure, Diversity, Tropical group, Temperate group, Breeding

## Abstract

**Background:**

Maize breeding germplasm used in Southwest China has high complexity because of the diverse ecological features of this area. In this study, the population structure, genetic diversity, and linkage disequilibrium decay distance of 362 important inbred lines collected from the breeding program of Southwest China were characterized using the MaizeSNP50 BeadChip with 56,110 single nucleotide polymorphisms (SNPs).

**Results:**

With respect to population structure, two (Tropical and Temperate), three (Tropical, Stiff Stalk and non-Stiff Stalk), four [Tropical, group A germplasm derived from modern U.S. hybrids (PA), group B germplasm derived from modern U.S. hybrids (PB) and Reid] and six (Tropical, PB, Reid, Iowa Stiff Stalk Synthetic, PA and North) subgroups were identified. With increasing K value, the Temperate group showed pronounced hierarchical structure with division into further subgroups. The Genetic Diversity of each group was also estimated, and the Tropical group was more diverse than the Temperate group. Seven low-genetic-diversity and one high-genetic-diversity regions were collectively identified in the Temperate, Tropical groups, and the entire panel. SNPs with significant variation in allele frequency between the Tropical and Temperate groups were also evaluated. Among them, a region located at 130 Mb on Chromosome 2 showed the highest genetic diversity, including both number of SNPs with significant variation and the ratio of significant SNPs to total SNPs. Linkage disequilibrium decay distance in the Temperate group was greater (2.5–3 Mb) than that in the entire panel (0.5–0.75 Mb) and the Tropical group (0.25–0.5 Mb). A large region at 30–120 Mb of Chromosome 7 was concluded to be a region conserved during the breeding process by comparison between S37, which was considered a representative tropical line in Southwest China, and its 30 most similar derived lines.

**Conclusions:**

For the panel covered most of widely used inbred lines in Southwest China, this work representatively not only illustrates the foundation and evolution trend of maize breeding resource as a theoretical reference for the improvement of heterosis, but also provides plenty of information for genetic researches such as genome-wide association study and marker-assisted selection in the future.

**Electronic supplementary material:**

The online version of this article (doi:10.1186/s12864-016-3041-3) contains supplementary material, which is available to authorized users.

## Background

Maize (*Zea mays* L.), which is one of the most widely grown crop in the world, and exceeds wheat and rice in production, plays an essential role in global food security [1]. With an increasing demand for maize, raising its production is an urgent challenge today. Because maize is an open-pollinated species with a complex genome [2], there is tremendous genetic diversity (GD) in the maize genome and it is considered a major factor in heterosis [3]. From the 1930s to the present, three stages in maize hybrid breeding history have been defined according to the source of parents: (1) Inbred lines directly derived from landraces during the 1930s–1950s; (2) Inbred lines derived from crosses among artificial selected inbred lines during the 1950s–1980s and (3) Inbred lines developed from cultivating elite inbred lines for commercial use [4]. Modern commercial breeding programs have brought about more than six-fold greater grain yields than those in previous decades [5]. However, owing to the number of valuable loci targeted during artificial selection, the GD of maize has gradually narrowed during the breeding process [6].

In heterosis, a hybrid offspring shows superior performance to the parents. At the genetic level, it is contributed by variation in the presence of genes or novel beneficial alleles and gene expression modification [7]. Theoretically, to maximize heterosis in maize, two inbred lines separated by a large genetic distance are selected as parents for commercial hybrids. Thus, identification of heterotic groups and patterns is the foundation of hybrid breeding. Previously, breeders assigned different inbred lines to specific groups using testcrosses, pedigrees and morphological traits. However, it is difficult to assign groups definitely by relying only on empirical information, particularly for lines with similar phylogenic background and complex sources. For this reason, molecular markers are widely used for this purpose. Application of molecular markers has undergone three main stages, from tens of restriction fragment length polymorphism markers (RFLPs) to hundreds of SSRs to millions of SNPs. Currently, SNPs are widely used because of efficient cost and high throughput [8].

During the last 30 years, many studies have focused on worldwide maize germplasm diversity. An early study used RFLPs to assign 148 inbred lines to two main groups, Iowa Stiff Stalk Synthetic (BSSS) and Lancaster, represented by B73 and Mo17, respectively [9]. Then, another study genotyped 94 SSRs to separate 260 inbred lines into four groups: non-Stiff Stalk (NSS), Tropical or Semitropical (TS), Stiff Stalk (SS) and a mixed group. TS group showed the highest GD [10]. In a large scale analysis, 96 SSRs were used to genotype 964 individuals representing almost the entire set of ~350 races native to the Americas. The entire panel was divided into four main clusters: highland Mexican, northern United States (U.S.), tropical lowland, and Andean races. The analysis showed that the southwestern U.S. was a transition area between Mexico and the northern U.S. [11]. In a later study, using the GoldenGate SNP chip, 770 inbred lines from CIMMYT, China and Brazil were clustered into eight groups, covering temperate and tropical germplasm and a special Chinese group Sipingtou (SPT) [12]. More recently, a new and low-cost SNP-genotyping technology named genotyping by sequencing (GBS) was used to genotype 2,815 maize inbred accessions in the U.S. seed bank. The results showed that the international germplasm pools were different from those commonly used in North America [13].

Maize was introduced into China approximately 500 years ago. Despite the narrow genetic background, there are two reasons for the complexity of Chinese germplasm: (1) Different germplasm was introduced into different areas and in different periods, and new germplasm is introduced continuously and (2) Chinese breeding programs have always integrated landraces and introduced germplasm and there is no complete record of this process [14]. For these reasons, systematic study of Chinese germplasm resources is important, particularly in the era of high-throughput SNP genotyping. Chinese inbred lines are classified into four to six heterotic groups [12], generally including Reid, SPT, and Lancaster as major groups according to the results of molecular characterization and pedigree [4,﻿ 12, 14–17]. Other new groups, such as the P (US hybrid P78599) group also known as PB group, derived from Pioneer hybrid P78599, have been clustered in most of studies [4,﻿ 12, 16, 17]. Using 70 SSRs and 1,034 SNPs, 187 and 282 Chinese inbred lines were grouped into six same clusters: PA, PB, Luda Red Cob (LRC), BSSS, Lancaster and SPT almost at the same time, respectively [12, 17]. Recently, using the 56 k MaizeSNP50 BeadChip, 1,015 SNPs were screened to classify 367 inbred lines into five subgroups containing P and Tem-tropic I in addition to the three major groups [4]. However, given that tropical germplasm and landraces for commercial hybrids are widely used in Southwest China because of a variety of breeding goals that contribute to a large different series of inbred lines comparing to other regions, thus these divisions of heterotic groups above are not sufficient for practical relevance.

With the development of high-throughput SNP genotyping, genome-wide association studies (GWAS) are widely used for gene mapping of complex quantitative traits. However, besides population structure, which is considered an essential factor resulting in false-positive estimation, linkage disequilibrium (LD) also determines the resolution of GWAS results by the decay distance corresponding to the marker coverage [18]. More rapid LD decay corresponds to shorter LD decay distance, requiring high marker density to cover functional loci and achieve high resolution for association mapping. In maize, the LD decay distance varies from less than 1 kb in landraces to more than 100 kb in commercial inbred lines [19]. Moreover, different populations and different segments of chromosomes always show varying LD. Current genome marker coverage is much higher than the previous coverage and allows fine estimation of LD decay distance in the genome-wide profile. For example, in a study using a 1536-SNP array to analyze LD decay distance in 632 lines from temperate, tropical, and subtropical breeding programs, the LD decay distance among 10 chromosomes ranged from 1 to 10 kb [20]. In a comparison of temperate, tropical, and subtropical lines, the LD decay distance in temperate lines was much higher than that of tropical and subtropical lines, owing to lower diversity and less rare alleles [21]. Recently, up to 681,257 SNPs were used for GWAS in 384 inbred lines from the Ames panel. The LD decay distance of that was close to 10 kb [22]. It implies that different degrees of panel diversity, marker density, and methods contribute to the variation in LD decay distance estimates.

However, previous studies have focused on population structure and GWAS in global germplasm, but for Southwest China, the complex ecological conditions in maize production contribute to the different germplasms used in this region so that pedigree of some local and new cultivated inbred lines are not clear. Besides, for the excellent resistance to the low soil fertility, severe disease caused by high temperature, wet, and less sunlight during maize growing period, the widespread use of tropical and semi-tropical lines for hybrids is the greatest characteristic in this region. As a consequence, these aspects contribute to the complexity in the construction of heterotic groups and heterotic pattern. For the present study, 362 diverse inbred lines widely used in Southwest China were collected for genotyping with the MaizeSNP50 BeadChip. Systematic characterization of germplasm including hererotic group clustering, GD comparison, and LD estimation was performed for the purpose of characterization of breeding resources and simplification of heterosis models in Southwest China.

## Methods

### Plant materials

A total of 362 diverse inbred lines from the current Southwest China breeding program were assembled into a panel. The panel was comprised of 33 parents of major expanded hybrids from Southwest China, 252 new selected and improved inbred lines, 40 representative inbred lines of temperate heterotic groups (Reid, SPT, Lancaster, LRC, etc.) and 37 CIMMYT or U.S. exotic inbred lines. Approximately 17 pairs of inbred lines were parents of local varieties and most of domestic inbred lines were one parent of authorized hybrids in China. Names and pedigree information for this panel are presented in Additional file 1: Table S1.

### SNP genotyping

A total of 56,110 SNPs on the MaizeSNP50 BeadChip were used for genotyping the panel. Detailed information for this chip can be downloaded from the Illumina MaizeSNP50 website [23] and the position information of SNPs according to B73 RefGen_v2 can be downloaded from NCBI (National Center for Biotechnology Information) GEO website [24]. One month after seed germination, eight leaf pieces from different individuals of the same inbred line were placed into one tube, and two replicates were collected for DNA extraction and genotyping. Using Illumina GenomeStudio, a powerful genotyping tool, each SNP was rechecked manually to identify any errors following Yan [8]. Then the raw file exported by GenomeStudio was transformed to PowerMarker V3.25 [25] format for calculation of summary statistics. SNPs with missing rate >20 %, heterozygosity >20 % and minor allele frequency (MAF) <0.05 were excluded from the genotyping data, leaving 44,104 high-quality SNPs for further analysis.

### Structure analysis, principal component analysis, and kinship matrix

To infer the population structure, further screening of SNPs from the 44,104 high-quality SNPs was performed according to a more stringent standard (missing rate <0.05, GD >0.45). A subset of 13,021 diverse SNPs were imported into STRUCTURE 2.3.4 [26], a Bayesian Markov chain Monte Carlo program for assigning individuals to groups. The number of subgroups (K) ranged from 1 to 10, and five times simulations with iterations and burn-ins set to 10,000 were performed using the mixture model and correlated allele frequency for each K. Based on the output log likelihood of data (LnP(D)) of STRUCTURE, the ad hoc statistic *ΔK* was used to determine the optimal number of subgroups [27]. Results of five replicate files were integrated using the CLUMPP software [28]. The threshold of possibilities of assigning individuals to corresponding groups depended on the breeding background particularly the representative inbred lines.

A total of 43,735 SNPs with identified physical positions were extracted from the 44,104 high-quality SNPs and further transformed to Hapmap format for analysis with TASSEL 5.2.10 [29]. According to the results of STRUCTURE, the principle component analysis (PCA) output data were visualized for each subgroup.

Finally, relative kinship coefficients were calculated using the Genomic Association and Prediction Integrated Tool (GAPIT) [30] with the same SNP subset used for PCA. Default algorithm VanRaden [31] was selected to calculate kinship.

### Genetic diversity analysis and phylogenetic tree

Two important parameters of diversity, GD and polymorphism information content (PIC), were estimated for all 56,110 SNPs using PowerMarker. GD is defined as the probability that two randomly chosen alleles from the population are different. It can be estimated at the *l*th locus as:

$$ {\widehat{D}}_l=\left(1 - {\displaystyle \sum_{u=1}^k}{{\tilde{P}}_{lu}}^2\right)/\left(1 - \frac{1+f}{\mathrm{n}}\right) $$

In which *f* represents the inbreeding coefficient, ${\tilde{P}}_{lu}$ the frequency of the *u*th allele, and n the sample size. PIC was estimated by:

$$ {\widehat{\mathrm{PIC}}}_l=1 - {\displaystyle \sum_{u=1}^k}{{\tilde{P}}_{lu}}^2 - {\displaystyle \sum_{u=1}^{k-1}}{\displaystyle \sum_{v=u+1}^k}2{{\tilde{P}}_{lu}}^2{{\tilde{P}}_{lv}}^2 $$

Where ${{\tilde{P}}_{lu}}^2$ and ${{\tilde{P}}_{lv}}^2$ are the frequency of the *u*th and *v*th alleles of marker *l*. To compare the GD among different groups, the GD and PIC value of 44,104 high-quality SNPs were calculated for each subgroup. The mean GD of SNPs in each 10-Mb bin was also calculated for the entire panel, Temperate group, and Tropical group according to the SNP physical position. Bins with <30 SNPs were dropped from this analysis because of limited statistical power. The average GD of SNPs in each bin was then calculated for the entire panel and for Temperate and Tropical groups. The distance to the centromere was also considered as an essential factor, because most low GD hotspots were close to the centromere region.

To construct a phylogenetic tree, 13,021 SNPs used in the STRUCTURE analysis were reloaded into PowerMarker. Nei’s genetic distances based on allele frequency were calculated, and a visual plot of the neighbor-joining (NJ) tree containing group cluster information was drawn with Dendroscope V3.2.10 [32].

### Allele frequency

The allele frequency, which is considered to play an important and basic role in explaining genetic differences, was compared between the Tropical and Temperate groups. To determine the variation among groups, the frequency of each allele was separately calculated for each SNP and compared between the Temperate and Tropical groups. The unique SNPs, defined only one of alleles existed in one of these groups above but not did in the other group, were screened in both groups. Moreover, SNPs with significant variation of allele frequency were identified by the χ^2^ test. The significance level was set to 0.001. Then, to investigate the overall allele frequency variation in the whole genome, the ratio of the number of SNPs with significant variation to the total number of SNPs was calculated for each 10-Mb bin based on SNP physical position.

### LD analysis

The LD between each pair of SNPs on each chromosome was evaluated by the squared Pearson correlation coefficient (r^2^). The cutoff of r^2^ was 0.1, and the LD decay distance was measured as the interval with an r^2^ value less than 0.1. A set of 43,735 SNPs with identified physical positions were loaded into TASSEL 5.2.10 for LD analysis. The LD decay distances were calculated separately for the entire panel and for the Temperate and Tropical groups.

### Pairwise comparison between inbred lines

To investigate individual variation at the whole-genome level, each pair of inbred lines in the panel was compared with respect to the alleles of each SNP. For each SNP, 0 referred to different alleles of this SNP between two individuals or, alternatively, 1 referred to the same allele of this SNP was common to the two individuals. The ratio of the sum of the counts to the total SNP number, called the similarity ratio, was calculated for each pair of inbred lines to characterize the similarity of the pair. Representative inbred lines were selected according to their similarity ratios. A comparison with the representative inbred lines was performed for each 10-Mb bin across the whole genome to identify diverse or conserved regions of the genome.

## Results

### Summary description of this panel

**Fig. 1:**
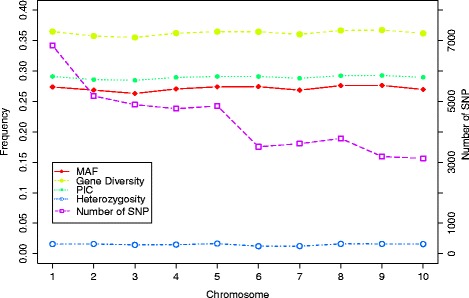
Summary statistics of 43,735 SNPs used for genotyping 362 inbred lines

After filtering of the total SNP data, 43,735 high-quality SNPs with identified physical position were used for summary description. In this subset of SNPs, the average marker density is approximately 52 kb per SNP, the average distance between adjacent SNPs is 47 kb, and the position of each SNP is listed in Additional file 2: Table S2. The MAF, GD, PIC value, and heterozygosity among chromosomes showed little variation among chromosomes by PowerMarker (Fig. 1). However, the numbers of SNPs showing significant variation, from 3128 on Chromosome (Chr) 10 to 6834 on Chr 1 were correlated with the lengths of the corresponding chromosomes. MAF was uniformly distributed from 0.1 to 0.5 among SNPs, but the trends of GD and PIC were similar, with most SNPs showing high levels of these parameters (48 % showing GD >0.45 and 55 % showing PIC >0.35) (Additional file 3: Figure S1).

### Population structure and kinship

**Fig. 2:**
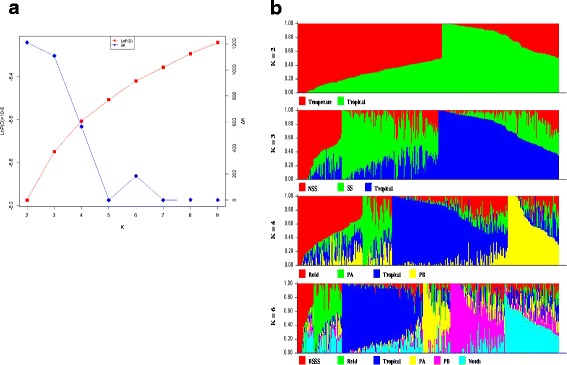
Population structure of 362 maize inbred lines estimated from 13,021 SNPs. **a** Plot of LnP(D) and *ΔK* was calculated for K = 2 to K = 9. **b** Population structure of the 362 lines from K = 2 to K = 4 and K = 6

STRUCTURE software was used to identify population structure. Because LnP(D) continuously increased with the K value, it was not capable of identifying groups. Accordingly, the *ΔK* value was calculated for each K. The peaks of line plot (Fig. 2a) suggested that the entire panel could be divided into two, three, four, six groups in order of possibility based on breeding experience and pedigree information. When K = 2, the whole panel could be grouped into Tropical and Temperate groups which the representative inbred lines were S37 and 18–599; When K = 3, the Temperate group was further divided into SS and NSS groups, including representative inbred lines Qi319 and Ye478, respectively; When K = 4, PA and PB were further separated which the representative inbred lines were 698–3 and 18–599; When K = 6, besides the Tropical subgroup, other inbred lines could be clustered into BSSS, Reid, PA, PB and North group (other temperate germplasms mostly derived from North China including Lancaster, SPT and LRC), the representative inbred lines were B73, Ye478, 698–3, 18–599, and Dan340, respectively. These results were largely consistent with known pedigree information. Other lines with lower possibility were clustered into a mixed group.

**Fig. 3:**
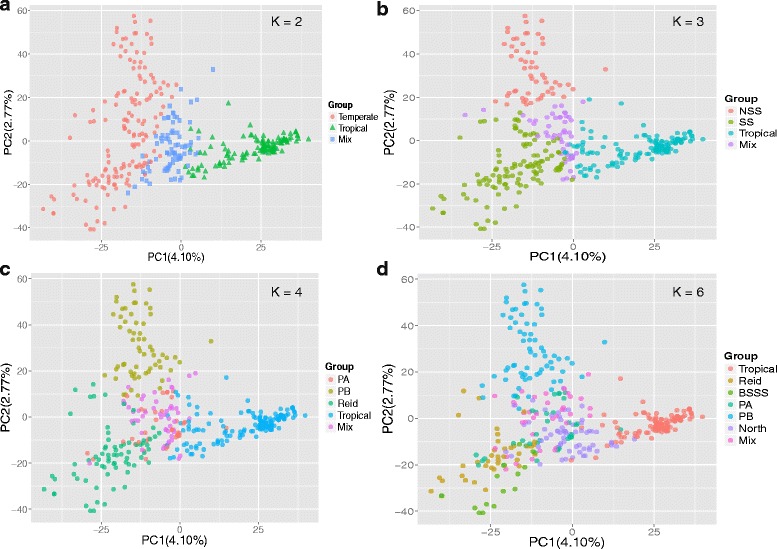
PCA plots for the entire panel and colored by the group divisions according to results of STRUCTURE in different K values. **a**–**d**: PCA plots for K values 2, 3, 4, and 6

PCA analysis based on the results of STRUCTURE showed a clear structure separation of the panel. When K = 2, the PCA plot showed a clear separation of Temperate and Tropical groups (Fig. 3a). The mixed group was located between the other two groups. The Temperate group showed more dispersion than the Tropical group did, suggesting that the temperate lines could be divided into further subgroups. For K = 3, the NSS and SS groups were clearly separated from the Temperate group and the genetic distance between SS and NSS was less than that between NSS and Tropical (Fig. 3b). For K = 4, the PA group was located at the center, mixed with other groups, while the other groups showed relatively stable distribution (Fig. 3c). For K = 6, the PCA plot showed a complex distribution of subgroups, except for the PB and Tropical groups. The BSSS group was largely from the Reid group, and the PA and North groups located at the center, showed a complex source (Fig. 3d). These results also agreed with the conclusions from STRUCTURE that the groups were gradually separated based on one main group, Temperate, with K increasing and the Tropical group remaining relatively stable during this process. To confirm the separation between the groups from the Temperate group, PCA analysis was also performed after removal of the Tropical and Mixed groups for K = 3, K = 4, and K = 6 (Additional file 4: Figure S2). Clear separations of the Temperate group were showed for K = 3 and K = 4, but more complex clustering for K = 6.

**Fig. 4:**
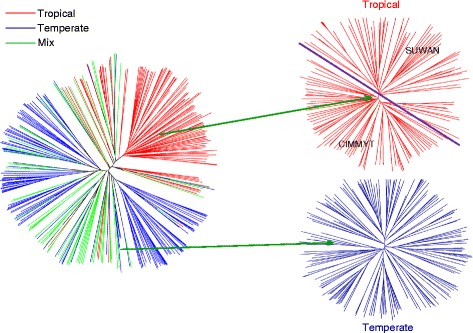
Phylogenetic tree of the entire panel for K = 2 and the subdivision of Tropical and Temperate group

Based on Nei’s genetic distance, the NJ phylogenetic tree also displayed a similar division of Tropical and Temperate among inbred lines of the whole panel (Fig. 4). Two clear branches representing Tropical and Temperate showed large differences and clear separation, whereas mixed inbred lines showed crossovers among this tree, but were intensively located at the transitional region between the two groups mentioned above. For tropical germplasm, the phylogenetic tree also showed a separation between two known introduced groups, Suwan and CIMMYT. However, with the increasing of K, the Temperate group also showed a complex and transitional process with minor differences that consistent with the results of PCA analysis (Additional file 5: Figure S3).

**Fig. 5:**
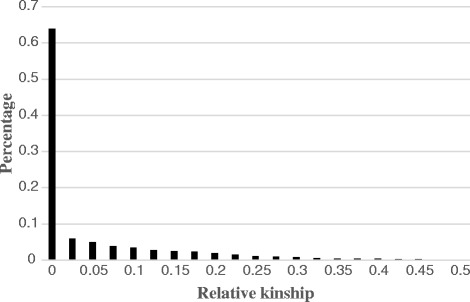
Distribution of pairwise kinship between 362 inbred lines. Only percentage of kinship estimates ranges from 0 to 0.50 are shown for simplicity

Relative kinship was estimated by a bar plot of the output kinship matrix (Fig. 5). 63.9 % of paired relative kinship estimates were equal to zero. 98.6 % of estimates were concentrated between 0 and 0.5, only a few estimates showed very close relationship around 2.0 (Additional file 6: Table S3). Overall, the kinship analysis indicated a relatively weak relationship among inbred lines of the whole panel.

### Genetic diversity

GD was estimated for the entire panel and each group. The entire panel showed the highest diversity (0.362), ranging from 0.095 to 0.500. In contrast, the BSSS group showed the lowest diversity (0.258) among groups. The Tropical group always showed higher GD (0.347, 0.352, 0.353, and 0.338) than Temperate group (0.331) in the overall K values, although the sample size was smaller. The highest GD among different K values for the Tropical group was 0.353 for K = 4. The GD of SS (0.341) was much higher than that of NSS (0.265). In the comparison of the PA and PB groups with K = 4 and K = 6, the PA group was more diverse than the PB group (0.284 to 0.276) but was much lower than the Reid group. Remarkably, for K = 6, the North group was more diverse than Tropical germplasm, with a high value of GD 0.355 (Table 1, Additional file 7: Figure S4).

**Table 1:** Genetic diversity and PIC of different groups for different K values

Group	Number of lines	Gene diversity	PIC value
Temperate	152	0.331 (0.000–0.500)	0.265 (0.000–0.375)
Tropical (K = 2)	127	0.347 (0.000–0.500)	0.278 (0.000–0.375)
Tropical (K = 3)	143	0.352 (0.000–0.500)	0.282 (0.000–0.375)
Tropical (K = 4)	142	0.353 (0.000–0.500)	0.282 (0.000–0.375)
Tropical (K = 6)	109	0.338 (0.000–0.500)	0.271 (0.000–0.375)
NSS	55	0.265 (0.000–0.500)	0.216 (0.000–0.375)
SS	114	0.341 (0.000–0.500)	0.272 (0.000–0.375)
Reid (K = 4)	72	0.326 (0.000–0.500)	0.261 (0.000–0.375)
Reid (K = 6)	37	0.286 (0.000–0.500)	0.233 (0.000–0.375)
BSSS	16	0.258 (0.000–0.500)	0.209 (0.000–0.375)
PA (K = 4)	36	0.287 (0.000–0.500)	0.232 (0.000–0.375)
PA (K = 6)	35	0.281 (0.000–0.500)	0.228 (0.000–0.375)
PB (K = 4)	61	0.273 (0.000–0.500)	0.222 (0.000–0.375)
PB (K = 6)	66	0.278 (0.000–0.500)	0.226 (0.000–0.375)
North	63	0.355 (0.000–0.500)	0.283 (0.000–0.375)
Entire	362	0.362 (0.095–0.500)	0.289 (0.091–0.375)

**Fig. 6:**
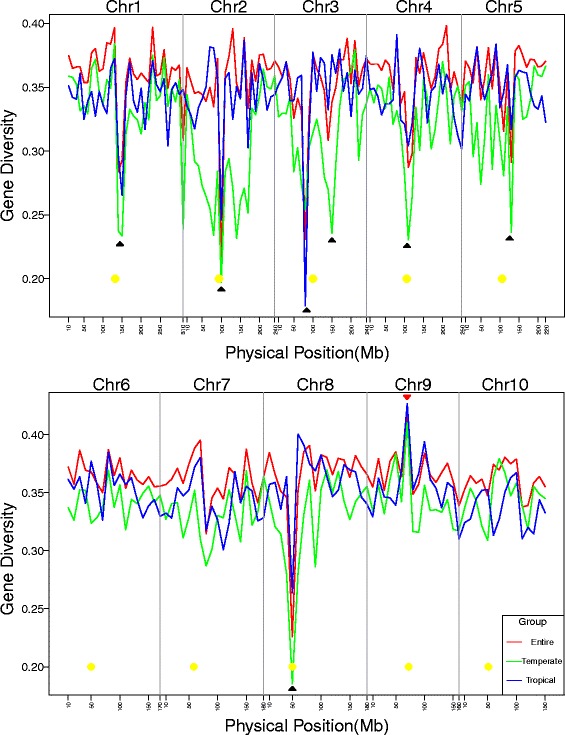
GD distribution of whole genome with 10-Mb bin size in three major groups. Yellow circles are centromere positions, black arrows indicate low-GD hotspots, and red arrows indicate high-GD hotspots

By calculating the GD of the entire panel, Temperate, and Tropical groups in 210 bins separated from the whole genome, the GD variation trends of the three groups were similar, but higher variation was detected in the Temperate group (Fig. 6). Seven significant and common low-GD hotspot regions (150 Mb in Chr 1, 100 Mb in Chr 2, 80 Mb and 150 Mb in Chr 3, 110 Mb in Chr 4, 130 Mb in Chr 5, 50 Mb in Chr 8) and one high-GD hotspot region (70 Mb in Chr 9) were identified with a cutoff (GD >0.40 for high hotspot and GD <0.25 for low hotspot) over the whole genome.

### Allele frequency analysis of Tropical and Temperate groups

After the comparison of the frequency of both alleles from each SNP, a total of 437 unique SNPs (See Methods) were screened from Tropical and Temperate groups (Table 2). In the Temperate group, 271 more unique SNPs were identified than in Tropical group, possibly accounting for the greater diversity of tropical group. Ten SNPs showing extreme differences in allele frequency between the two groups were also identified as a core set of markers for the distinguishing of the two groups (Table 3).

**Table 2:** Number of unique SNPs in the Temperate or Tropical group

Chromosome	Number of unique SNP in Temperate group	Number of unique SNP in Tropical group
1	41	20
2	41	6
3	55	7
4	39	14
5	26	5
6	17	5
7	35	8
8	46	10
9	26	1
10	25	7
Unknown	3	0
Total	354	83

**Table 3:** Ten SNPs with the highest allele frequency variation between the Tropical and Temperate groups

SNP name	Chr^a^	Position	Allele	Allele frequency		Allele frequency variation
				Tem^b^	Tro^c^	
PZE-103086995	3	144096829	A	0.888	0.118	0.770
SYN23575	2	49925995	G	0.855	0.094	0.761
PZE-103087179	3	144683803	A	0.066	0.795	0.729
PZE-103087189	3	144684150	C	0.066	0.795	0.729
SYN14294	2	152502748	A	0.178	0.906	0.728
PZE-103087178	3	144683738	C	0.908	0.181	0.727
SYN20183	3	144689036	G	0.914	0.197	0.717
PZE-103087199	3	144687159	C	0.066	0.780	0.714
SYN20185	3	144689964	G	0.066	0.780	0.714
SYN20186	3	144687048	C	0.066	0.780	0.714

^a^Chr, Chromosome; ^b^Tem, Temperate group; ^c^Tro, Tropical group

**Fig. 7:**
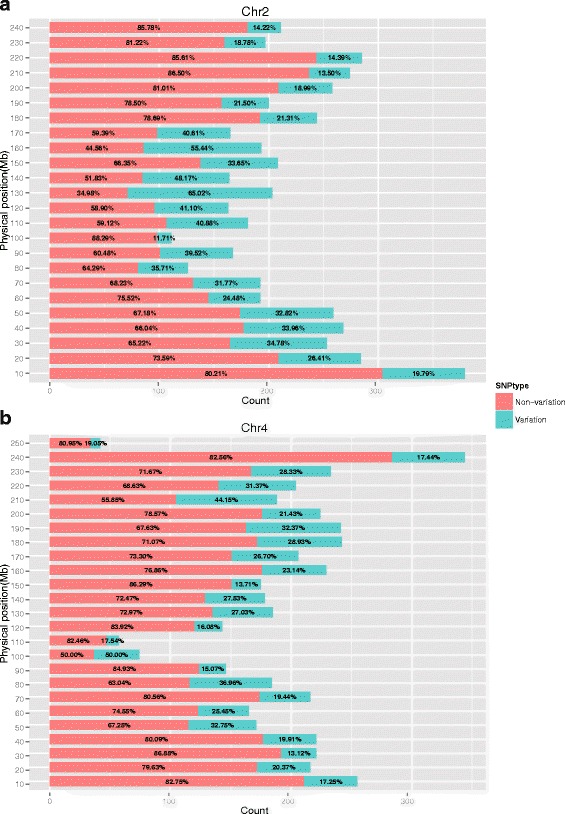
Distribution of variant SNPs and variant SNPs/total SNPs ratio of the two (Chr 2 and Chr 4) most diverse chromosomes by 10-Mb bin size. **a** Bar plot of variant SNPs count and variant SNPs/total SNPs ratio in Chr 2. **b** Bar plot of variant SNPs count and variant SNPs/total SNPs ratio in Chr 4. In each bar, percentage number on red part and cyan part represent non-variant SNPs/total SNPs ratio and variant SNPs/total SNPs ratio of the corresponding bin, respectively

At the chromosome level, 1531 variant SNPs and the ratio (0.296) of the number of variant SNPs to the number of total SNPs were both the highest on Chr 2, whereas Chr 10 showed the lowest values among the chromosomes (Additional file 8: Table S4). To reveal the distribution of SNPs with significant variation in allele frequency between the two groups, the whole genome was divided by the 10-Mb bins for further analysis. Variant and non-variant SNP number and the corresponding ratio (variant SNPs/total SNPs and non-variant/total SNPs) were also calculated (Fig. 7 and Additional file 9: Figure S5). One bin located at 130 Mb of Chr 2 with 132 variant SNPs and the variant SNPs/total SNPs ratio (65.02 %) held both the highest number of variant SNPs and highest variant SNPs/total SNPs ratio over the whole genome.

### Linkage disequilibrium decay distance

**Fig. 8:**
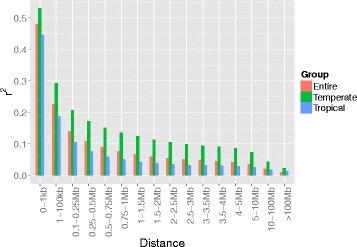
Mean r^2^ over different intervals of LD decay distance for the entire panel, Temperate, and Tropical groups

The average r^2^ of each distance interval was calculated for the estimation of LD decay distance (Fig. 8). At a cutoff of *r*^*2*^ = 0.1, the Tropical group showed the smallest LD decay distance (0.25–0.50 Mb) among all of the groups. The entire panel followed the Tropical group with an LD decay distance of 0.50–0.75 Mb. The Temperate group showed the largest distance (2.5–3 Mb). Among the chromosomes in the panel, Chr 1 showed the smallest distance and Chr 8 showed the largest. LD decay distance over ten chromosomes in the Tropical group always showed the smallest among the groups. Chr 1, 2, and 6 were similar at 0.1 to 0.25 Mb. In contrast, in the Temperate group, Chr 1 showed the smallest LD distance (1.5–2 Mb), and Chr 10 the largest up to 5–10 Mb (Table 4).

**Table 4:** LD decay distance of 10 chromosomes in the entire panel, Tropical and Temperate groups

Chromosome	The entire panel (Mb)	Tropical group (Mb)	Temperate group (Mb)
1	0.25–0.5	0.1–0.25	1.5–2
2	0.5–0.75	0.1–0.25	4–5
3	0.5–0.75	0.25–0.5	3.5–4
4	0.5–0.75	0.25–0.5	2–2.5
5	0.5–0.75	0.25–0.5	2.5–3
6	0.5–0.75	0.1–0.25	2–2.5
7	0.5–0.75	0.25–0.5	2–2.5
8	0.75–1	0.25–0.5	2.5–3
9	0.5–0.75	0.25–0.5	3–3.5
10	0.5–0.75	0.25–0.5	5–10
Total	0.5–0.75	0.25–0.5	2.5–3

### Pairwise comparison of entire panel

**Fig. 9:**
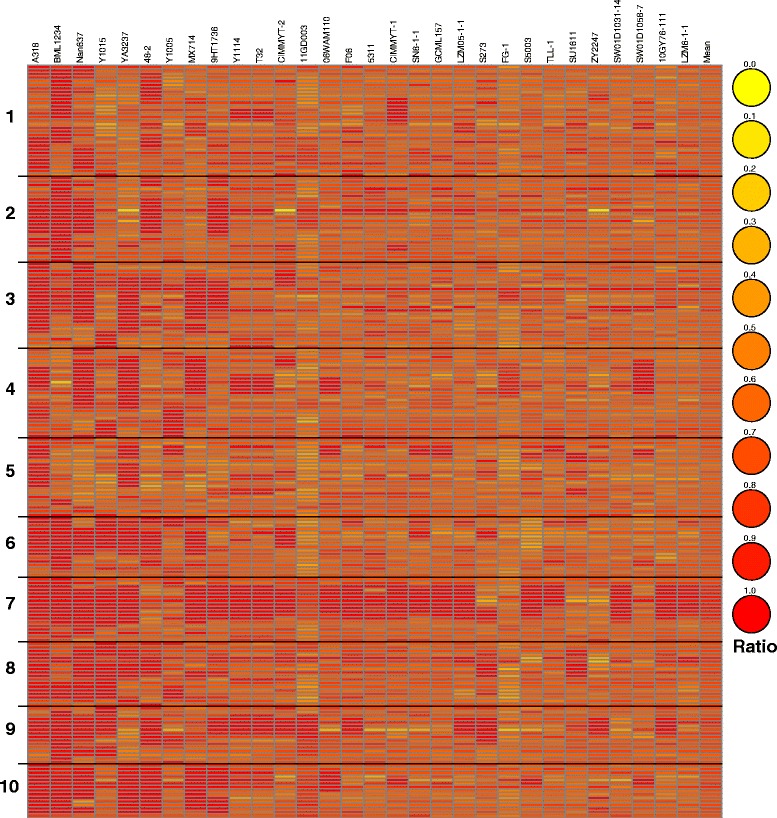
Heat map of similarity ratio of S37 to subset of 30 lines most similar to S37 by 10-Mb bin size across the whole genome. Numbers on the left represent chromosomes. Names of inbred lines are shown at the top. The width of each bar means a 10-Mb bin

Pairwise comparison was used to detect the similarity of each pair of lines in the panel (Additional file 10: Figure S6). For all of the pairs in the panel, the mean similarity ratio was 0.591. The highest rate was 0.993 between Zheng22 and U8112 and the lowest rate was 0.353 between Ji477 and 9HT1804 (Additional file 11: Table S5 and Additional file 12: Table S6). Moreover, as a superior and widely-used tropical inbred line in Southwest China, S37 and the 30 highest-similarity lines were screened as a subgroup to identify the high-similarity segments throughout the entire genome. Calculation of the average similarity to S37 for each chromosome revealed that each chromosome showed only a small difference from S37, varying between 0.683 in Chr 5 and 0.798 in Chr 7 (Additional file 13: Table S7). To investigate high-similarity segments in chromosomes, the similarity by a bin size of 10 Mb was also analyzed by heat map (Fig. 9). Several regions showing high similarity (>0.85) are listed in Table 5. A large region located in Chr 7 (30–120 Mb) that showed an average similarity of 0.888 was apparently conserved relative to S37 and will be valuable for future genomic analysis.

**Table 5:** Regions with a high similarity ratio (>0.85) in 30 inbred lines most similar to S37

Chromosome	Bin size	Total SNP Number	Mean
2	100	111	0.871
3	80	57	0.951
7	30	188	0.869
7	40	189	0.868
7	50	175	0.893
7	60	117	0.907
7	70	84	0.891
7	80	126	0.901
7	90	168	0.914
7	100	178	0.889
7	110	239	0.884
7	120	178	0.865
8	50	68	0.864
9	50	61	0.883

## Discussion

### Population structure showed gradual simplification with decreasing of K value

The 56 K SNP BeadChip was successfully used for genotyping diverse inbred lines from Southwest China. This is the first systematic large-scale study of heterotic groups of lines from Southwest China using the high-throughput SNP chip.

According to *ΔK* values of STRUCTURE, different numbers of groups were divided into two, three, four and six subgroups. Unlike most studies, which use only temperate lines to construct a population, the Tropical group was continuously present at all K values, demonstrating Tropical group is widely-used in Southwest China, and difficult to be subdivided. It is reasonable that the Tropical group includes most landraces with mixed complex pedigrees, and experiences only a short breeding time with few recombinations. In contrast, the Temperate group was divided clearly into several groups. Temperate lines were divided into two groups (SS, NSS), three groups (PA, PB, and Reid), and five groups (BSSS, Reid, PA, PB, and North). To characterize the difference in temperate group division from previous studies in other areas of China, a comparison was performed according to the results of two, three, four, and six groups. Most of the groups were found in the previous studies. This finding shows that the temperate germplasm has been widely used in southwest Chinese breeding programs. One group used in North China integrating several germplasm sources (Lancaster, SPT and Luda Red Bone) is a new group first identified in our study.

**Fig. 10:**
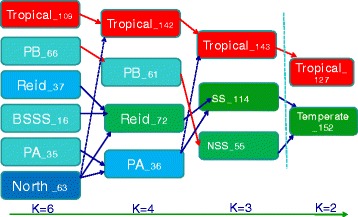
Pathway of population simplification from K = 6 to K = 2. Number following a group represents the group size. Red arrow represents the unique path from the initial population. Blue arrow represents the multiple major sources from the initial population. Dashed line arrow means a rare path from the last population. Mixed group are excluded for each K value

Owing to the use of large number of landraces and tropical lines in Southwest China, four different numbers of groups were identified in the panel showing more complex than other studies [4,﻿ 17]. The hererotic groups progressed from complex to simple with decreasing K value. The direction of lines was described from six groups to only the Tropical and Temperate groups (Fig. 10). This reflects the fine structure of the Temperate group in Southwest China, and further provides abundant information for the clear origin of each subgroup. PCA in the temperate lines showed clear and consistent separation among different K values (Fig. 3 and Additional file 4: Figure S2).

Because this panel covered most of widely used lines in Southwest China, these results can give us many theoretical references in breeding. The simplified division graph (Fig. 10) of Temperate group allows us to understand the foundation and evolution trend of breeding resources in this region. Furthermore, based on the current existed hybrids crossing information and the division of heterotic groups, the rules of heterotic pattern can be found as a guideline for the heterosis improvement and hybrid breeding.

### Genetic diversity in different groups

The average GD of the entire panel, Tropical group and Temperate group were 0.362, 0.348 and 0.331. Comparing with previous studies [4,﻿ 12, 33–36] (Additional file 14: Table S8), GD of the entire panel was higher than that of Lu et al. (2009) and Van Inghelandt et al. (2010) which was around 0.32, and was similar to that of Wu et al. (2014) and Wu et al. (2015) which were around 0.365, but was lower than that of Yang et al. (2011) which was 0.39. The variation in GD was strongly associated with the different populations. Furthermore, when the GD was compared across the different groups, the Tropical group always showed very high GD (>0.3) across different K values, a finding consistent with the results of previous results showing that the GD of tropical/subtropical germplasm is high [10]. In contrast, BSSS showed the lowest GD among all of the groups. Interestingly, the North group showed the highest GD, up to 0.355 when K = 6, probably owing to presence of a mixture of lines from different germplasms. On the other hand, SS group showed a much higher GD than the NSS group did, with both groups showing higher GD than PA or PB. Our study also showed that the GD is extremely variable across different regions. The centromere region always showed the lowest GD because only a few recombinations have occurred historically. In addition, regions in the entire panel showed significantly high or low GD in chromosomes displaying a similar trend in the Temperate and Tropical groups, but the variation of GD was more pronounced in temperate germplasm. The highest GD across the genome was found in bin Chr 9: 60–70 Mb, where the GDs of the entire panel, Temperate, and Tropical groups were all >0.40. The finding implies that this region has rarely undergone high selective pressure during the breeding process.

### Linkage disequilibrium decay distance

In our study, there was large variation in LD decay distance among the entire panel and the Tropical and Temperate groups. Higher LD decay distance of Temperate group comparing with that of the entire panel and Tropical group confirmed that Temperate group in China has undergone high selection pressure, a finding consistent with the GD results described above. At the chromosome level, LD decay distance also showed apparent variation, indicating that selection pressure in different chromosomal regions was varied because of different selection goals in breeding.

The average LD decay distance in our study (0.50–0.75 Mb) is similar to that (>500 kb) in a population of 192 elite inbred lines [37] and to that estimated for 285 diverse Dent inbred lines (approximately 500 kb) [38]. However, this value was slightly greater than the 391 kb observed in a population of 367 lines [4], and much longer than the LD decay distances of <100 kb [20, 21]. According to the LD decay distance was approximately 100 kb in commercial elite inbred lines in a previous study [19], 0.50–0.75 Mb is reasonable for many elite lines included in our panel. In summary, the LD decay distance is influenced by the source of inbred lines and selection intensity during the breeding process.

### Genetic impact of the introduction of tropical germplasm into Southwest China

Analysis of different genetic features above give us many clues to understand the genetic impact of tropical germplasm introducing into Southwest China. To population structure, tropical germplasm formed a large dependent subgroup, and more inbred lines were clustered into Mix group (83 lines when K = 2) (Additional file 1: Table S1). This also indicates many new cultivated lines were identified that contain part of tropical background for the adaption of local environment. To GD and LD decay distance, for the present of tropical germplasm which had higher GD and shorter LD decay distance than temperate germplasm, these of the entire population were respectively increased and reduced. As a consequence, increase of GD potentially provide breeders new alleles for selection and the reduction of LD decay distance can improve resolution of genetic analysis in this region.

### Comparison between the Tropical and Temperate groups

Previous studies found that the Tropical group had high diversity and short LD decay distance. Because the Tropical group is widely used in Southwest China, a systematic comparison of GD, LD, and allele frequency was performed between the Tropical and Temperate groups. The same conclusion that the Tropical group shows higher GD, shorter LD and less unique alleles was reached in our study. The number of variant SNPs and the allele frequency ratio of variant SNPs to total SNPs showed that Chr 2 contained the most variant SNPs and also showed the highest ratio among the chromosomes in the panel.

Modern elite inbred lines have experienced strong breeding selection. Because beneficial genes for agronomic traits were selected during this process, this has resulted in the reduction of their genetic diversity [39]. Non-genic regions also contribute to the reduced nucleotide diversity in the Temperate group [40]. Because the Temperate group has undergone a long period of artificial selection, it is reasonable that the Temperate group showed relatively low GD and short LD in its genome.

Allele frequency is a reflection of GD and can be affected by genetic drift and selection. For elite inbred lines, variation between the two groups is caused mainly by selection in different genomic regions. Another study has suggested that unique alleles provide biased estimates of GD, called “ascertainment bias”, because they are unable to show diversity in other groups [12]. Some SNPs in the array are were based on diversity within one group, possibly leading to SNP monomorphism in other groups. However, in our study, considering that a much larger-scale array (56,110 versus 1536) was used, there was only a small effect on GD and allele frequency estimation. To date, few studies have focused on the segments in the genome that differentiate breeding groups, possibly owing to large differences in the collection of lines for population construction. In our study, large segments and bins were all identified for the two major groups, and the next area of research is ascertaining the relationship with the collection of QTLs and SNP hits, particularly for traits differentiating the Tropical and Temperate groups such as flowering time and photoperiod.

### Pairwise comparison among lines

In this study, pairwise comparison effectively explained the relationship between pairs of randomly chosen lines. This method provides a simple and direct way to explore the relationship between the two lines. Comparison with the kinship matrix in the highest and lowest ten pairs of lines suggested that each pair of lines with high similarity consistently showed high kinship value (Additional file 11: Table S5 and Additional file 15: Table S9), findings that together are confirmed by breeding experience. Moreover, by defining different bin sizes according to the statistical power defined by SNP number, the whole genome can be divided into hundreds of bins such that similarity in small regions across chromosomes can be confidently detected. For instance, in the present study, S37 and other similar tropical lines were compared for identifying the conserved regions in the whole genome.

### Comparison of other studies using MaizeSNP50 BeadChip for population analysis

By now, MaizeSNP50 BeadChip are widely used in many studies such as evolution, population structure, GWAS and QTL fine mapping for its high density (greater than 25 markers per megabase according to B73 reference genome on average), high quality and wide source (Pazea, Syngenta and INRA) of SNPs. Although vast majority of SNPs are from the polymorphism between B73 and Mo17, and other 25 diverse lines, it still works well for the population analysis even in teosinte, the wild ancestor of maize. Comparing studies for population analysis using the same array [4﻿, 33, 38, 41–43] (Additional file 16, Table S10), the LD decay distance is ranged from 30 kb to 643 kb, and number of subgroups is from 2 to 7 which are reasonable because of the variance germplasm resources. Besides, regions showed differences between two subgroups or conservative in descendants of inbred lines were also identified in some studies. In our study, we not only identified the region enriched SNPs which showed significant difference between temperate and tropical germplasm, but also revealed the evolution trend of germplasm. So far, many biparental populations have been constructed by inbred lines from this panel. Taking advantage of high-density SNPs, more markers can be provided for QTL fine mapping. By combining with GWAS results, more convincing conclusions can be drawn which is a specific highlight to use this array in Southwest China.

## Conclusions

Using the MaizeSNP50 BeadChip, we performed the population structure, GD, and LD decay distance analysis of 362 important inbred lines collected from the breeding program of Southwest China for the first time. We identified two, three, four, and six subgroups according to the bioinformatic study and breeding experience. Tropical group showed higher GD and short LD decay distance comparing with the Temperate group. Highest GD region for both numbers of SNPs with significant variation and the ratio of significant SNPs to total SNPs were both located at 130 Mb region on Chr 2. A region at 30–120 Mb of Chr 7 was identified as a conserved region of S37 and most similar derived lines during the breeding process. This work representatively not only illustrates the foundation and evolution trend of maize breeding resources as a theoretical reference for the improvement of heterosis in this region, but also provides plenty of information for genetic researches such as genome-wide association study and marker-assisted selection in the future.

## Additional files

Additional file 1:
**Table S1.** Names, pedigree and co-ancestral membership of each inbred line in this panel. K = 2, 3, 4, and 6 mean the entire population can be clustered into 2, 3, 4 and 6 subgroups. (XLSX 61 kb) Additional file 2:
**Table S2.** Detailed information of 43,735 high-quality SNPs with identified physical position (B73 RefGen_V2). (XLSX 4226 kb) Additional file 3:
**Figure S1.** Frequency distribution of genetic diversity (**a**), MAF (**b**), and PIC (**c**) value among 362 maize inbred lines genotyped with 43,735 SNPs. (PDF 141 kb) Additional file 4:
**Figure S2.** PCA plot for the Temperate group at different K values according to the result of STRUCTURE. **a**–**c** show the PCA plots for K values 3, 4, and 6, respectively. (PDF 477 kb) Additional file 5:
**Figure S3.** Phylogenetic tree for different subgroups at K = 3, K = 4, and K = 6. (PDF 363 kb) Additional file 6:
**Table S3.** Pairwise kinship matrix of the entire population calculated by VanRaden algorithm. (XLSX 1849 kb) Additional file 7:
**Figure S4.** Box plot of genetic diversity for each subgroup at different K values (2, 3, 4, and 6). (PDF 170 kb) Additional file 8:
**Table S4.** Distribution of significant variant SNPs across 10 chromosomes (*p*-value < 0.001). (DOCX 13 kb) Additional file 9:
**Figure S5.** Distribution of variant SNPs and the ratio of variant SNPs/total SNPs in Chr 1, 3, 5, 6, 7, 8, 9, and 10 (**a**–**h**) by 10-Mb bin size. (PDF 425 kb) Additional file 10:
**Figure S6.** Heat map of pairwise comparison between lines in the whole panel. The color gradient from yellow to red represents similarity ratio from low to high. (PDF 660 kb) Additional file 11:
**Table S5.** Ten pairs of inbred lines with the highest pairwise similarity ratios among the entire panel. (DOCX 14 kb) Additional file 12:
**Table S6.** Ten pairs of inbred lines with the lowest pairwise similarity ratios among the entire panel. (DOCX 13 kb) Additional file 13:
**Table S7.** Similarity ratio of 30 inbred lines most similar to S37 for 10 chromosomes. (DOCX 18 kb) Additional file 14:
**Table S8.** Comparison of GD in different studies. MICN is an abbreviation of Modified introduction in China; TS is an abbreviation of Tropical/Subtropical; SS is an abbreviation of Stiff Stalk; NSS is an abbreviation of non-Stiff Stalk; HZS is an abbreviation of Huangzaosi. (XLSX 11 kb) Additional file 15:
**Table S9.** Ten inbred line pairs with the highest kinship coefficient among the entire panel. (DOCX 13 kb) Additional file 16:
**Table S10.** Comparison of other studies using the same SNP array for population analysis. MICN is an abbreviation of Modified introduction in China; TS is an abbreviation of Tropical/Subtropical; SS is an abbreviation of Stiff Stalk; NSS is an abbreviation of non-Stiff Stalk; HZS is an abbreviation of Huangzaosi. (XLSX 10 kb)

## Abbreviations

BSSS: Iowa Stiff Stalk Synthetic
Chr: Chromosome
CIMMYT: International Maize and Wheat Improvement Center
DNA: Deoxyribonucleic acid
GAPIT: Genomic Association and Prediction Integrated Tool
GBS: Genotyping by sequencing
GD: Genetic diversity
GWAS: Genome-wide association study
LD: Linkage disequilibrium
LnP(D): Log likelihood of data
LRC: Luda Red Cob
Mb: Megabase
NCBI: National Center for Biotechnology Information
NJ: Neighbor-joining
NSS: Non-Stiff Stalk
PA: Group A germplasm derived from modern U.S. hybrids
PB: Group B germplasm derived from modern U.S. hybrids
PCA: Principal component analysis
PIC: Polymorphism information content
RFLP: Restriction fragment length polymorphism
SNPs: Single nucleotide polymorphisms
SPT: Sipingtou
SS: Stiff Stalk
SSR: Simple sequence repeat (markers)
TS: Tropical or semitropical
U.S.: United States
