# Supplementary figures and images for: Characterizing the population structure and genetic diversity of maize breeding germplasm in Southwest China using genome-wide SNP markers
Source: BMC Genomics. 2016 Aug 31;17(1):697. doi: 10.1186/s12864-016-3041-3 (PMC5007717; doi:10.1186/s12864-016-3041-3)

**a**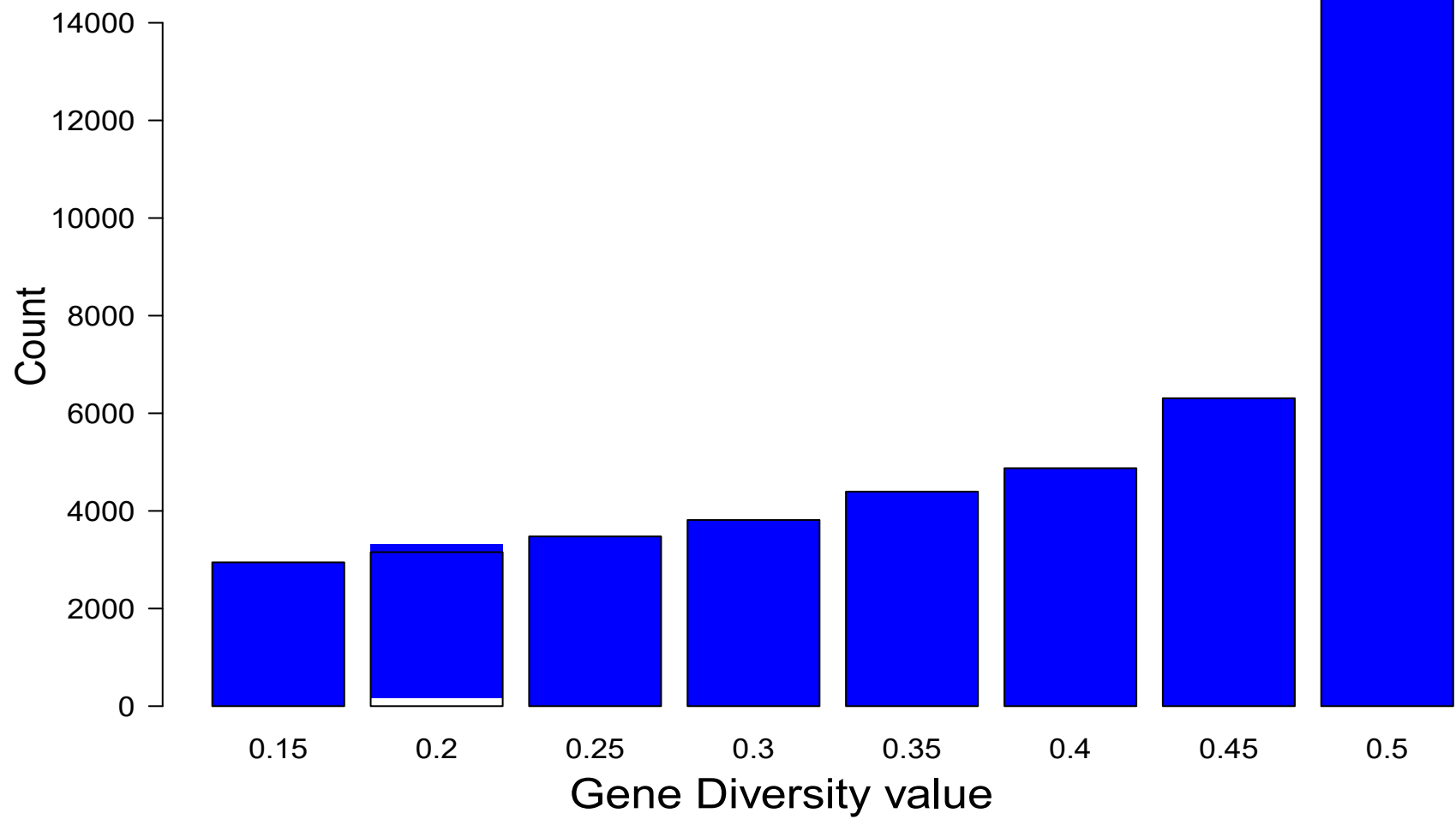**b**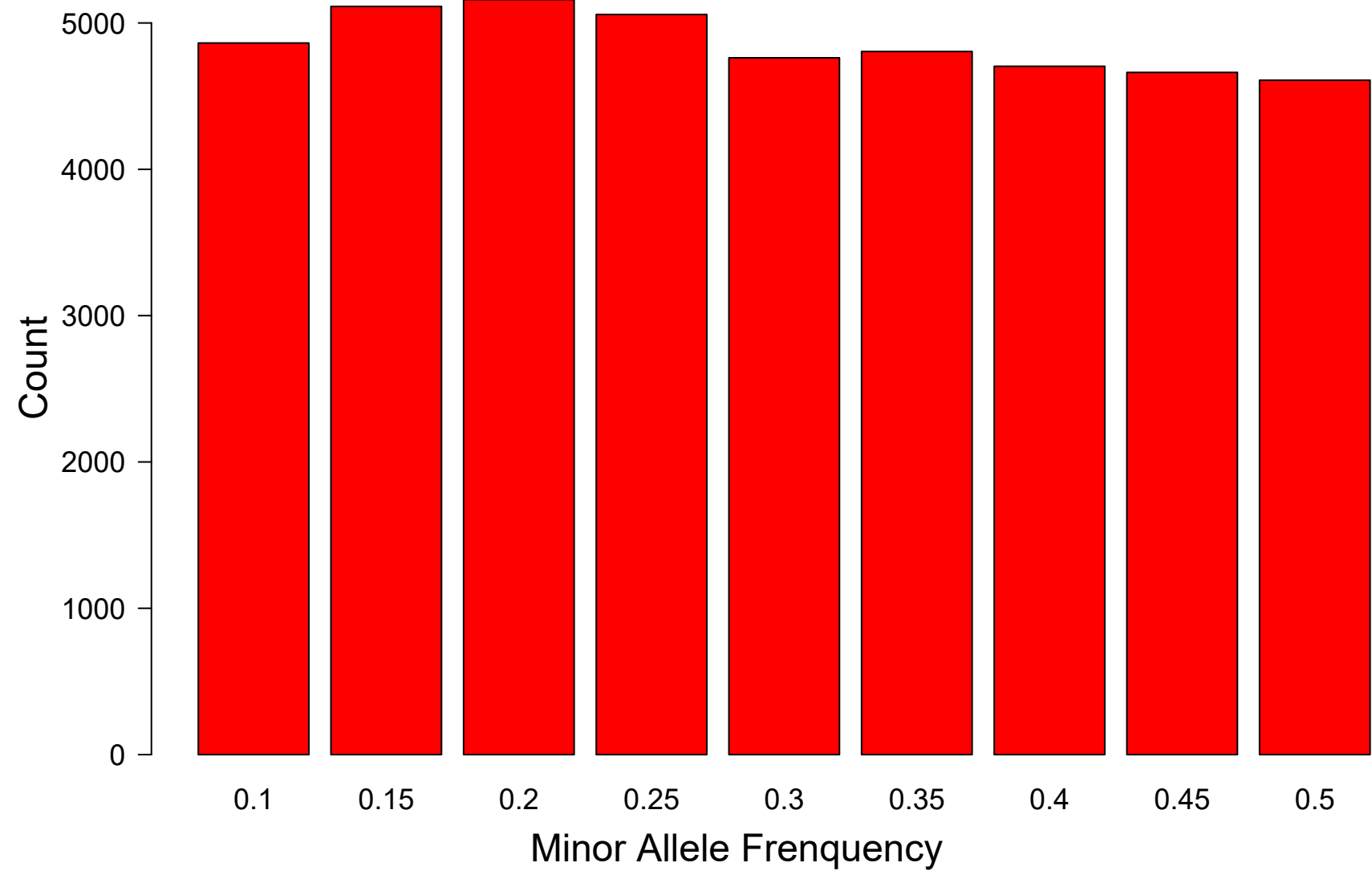**c**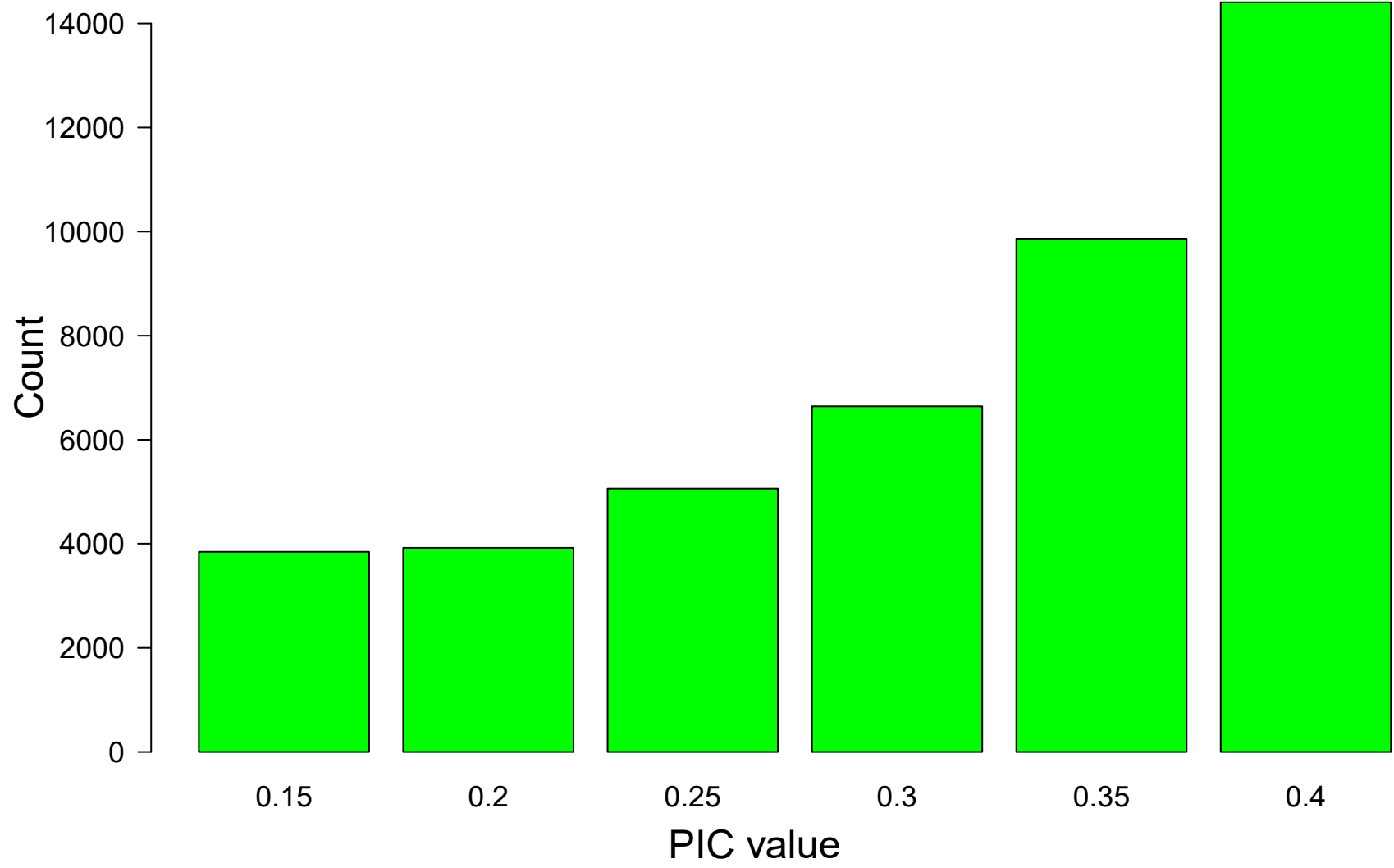

Supplement: Additional file 3: — Figure S1. Frequency distribution of genetic diversity (a), MAF (b), and PIC (c) value among 362 maize inbred lines genotyped with 43,735 SNPs. (PDF 141 kb) [file 12864_2016_3041_MOESM3_ESM.pdf]

**a**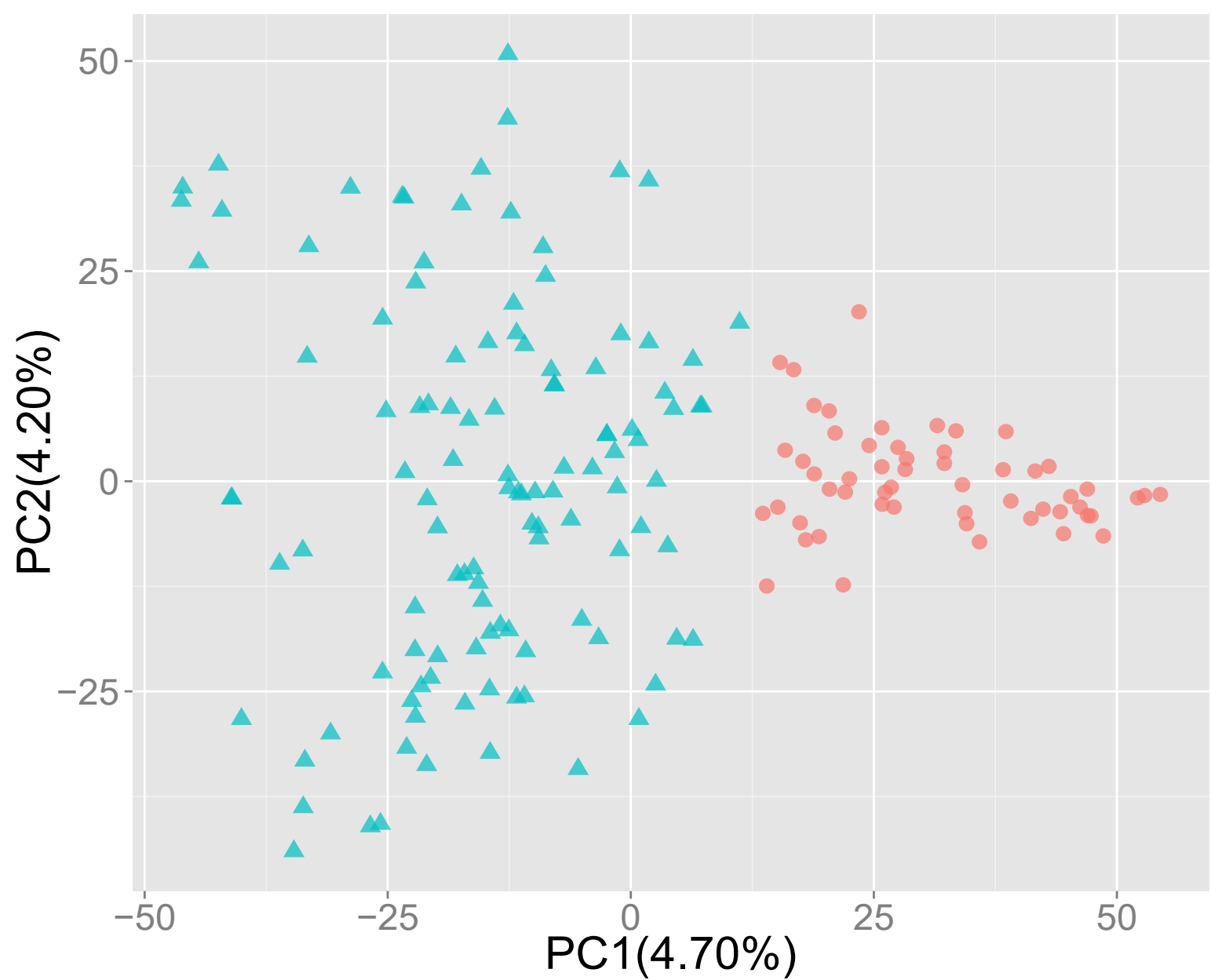**b**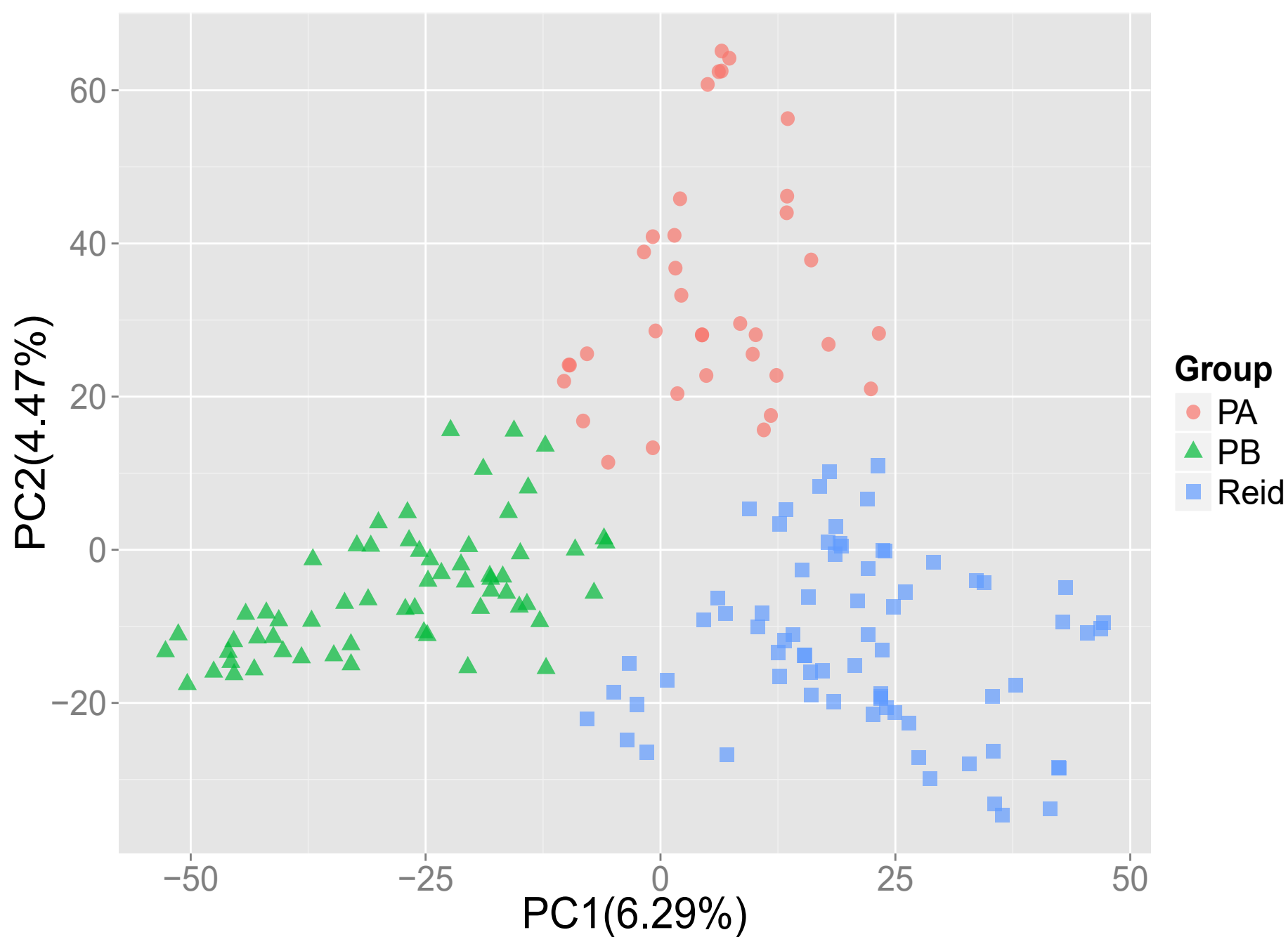**c**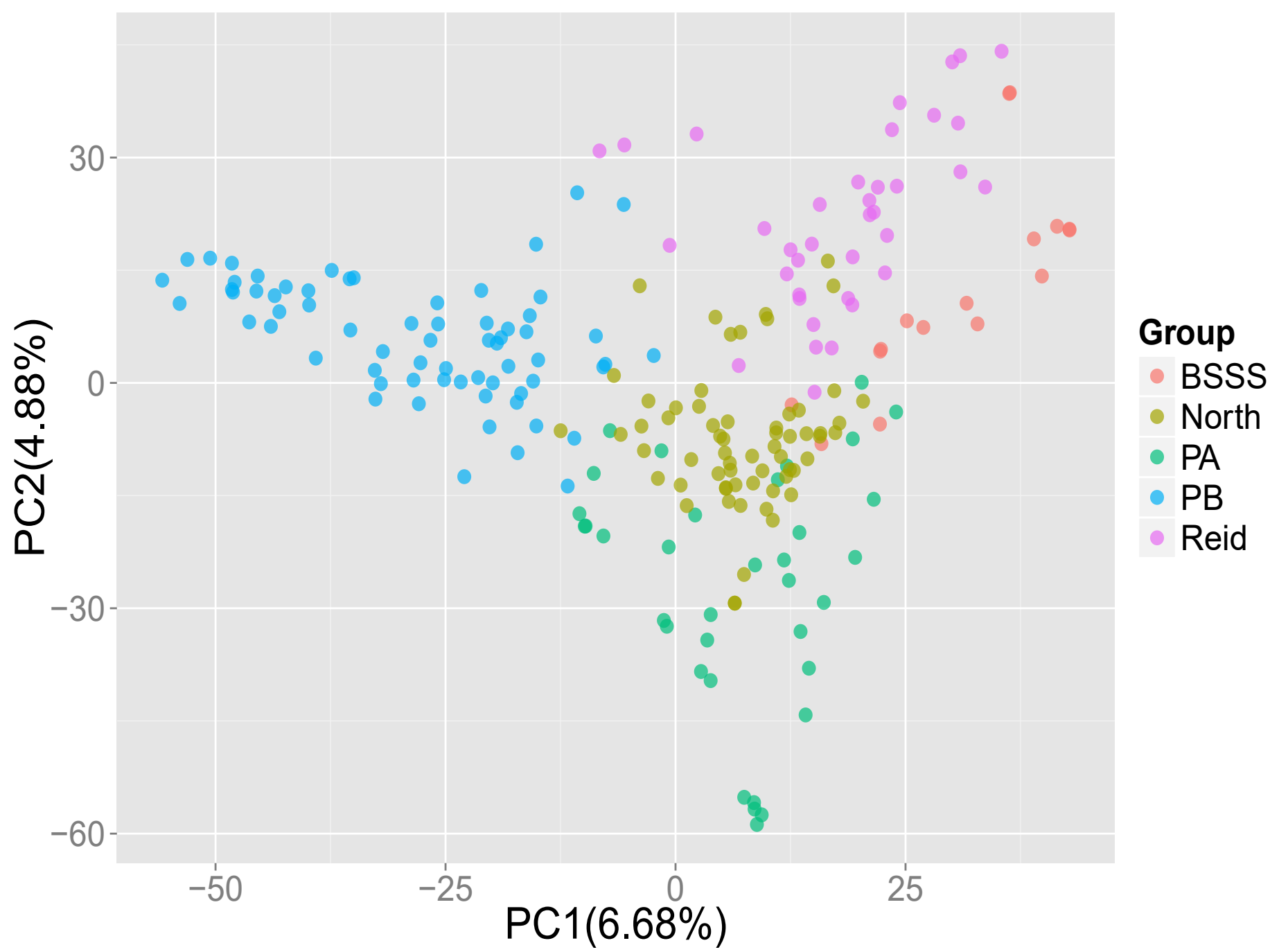

Supplement: Additional file 4: — Figure S2. PCA plot for the Temperate group at different K values according to the result of STRUCTURE. a–c show the PCA plots for K values 3, 4, and 6, respectively. (PDF 477 kb) [file 12864_2016_3041_MOESM4_ESM.pdf]

**a**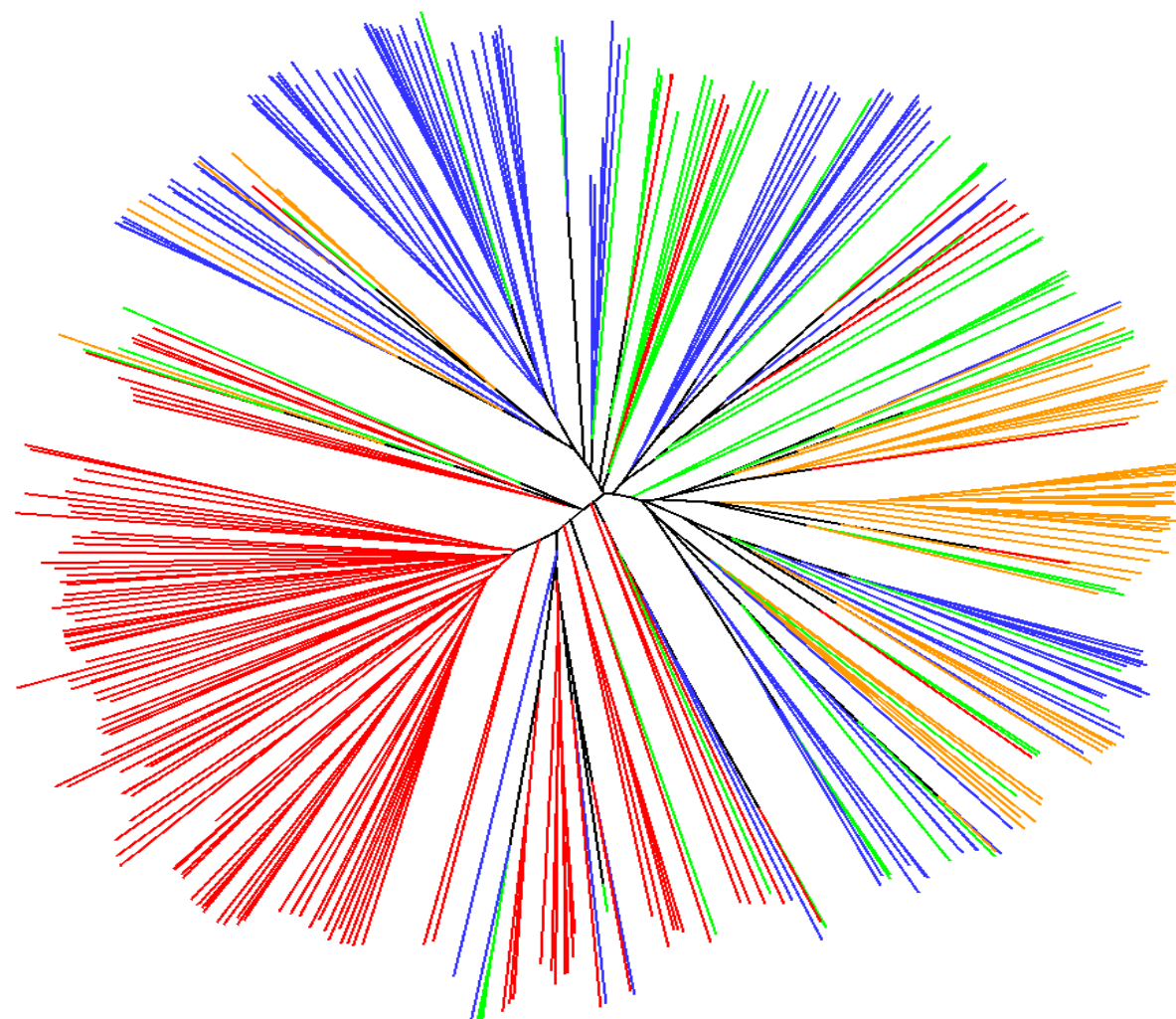

— Tropical  
— SS  
— NSS  
— Mix

K = 3

**b**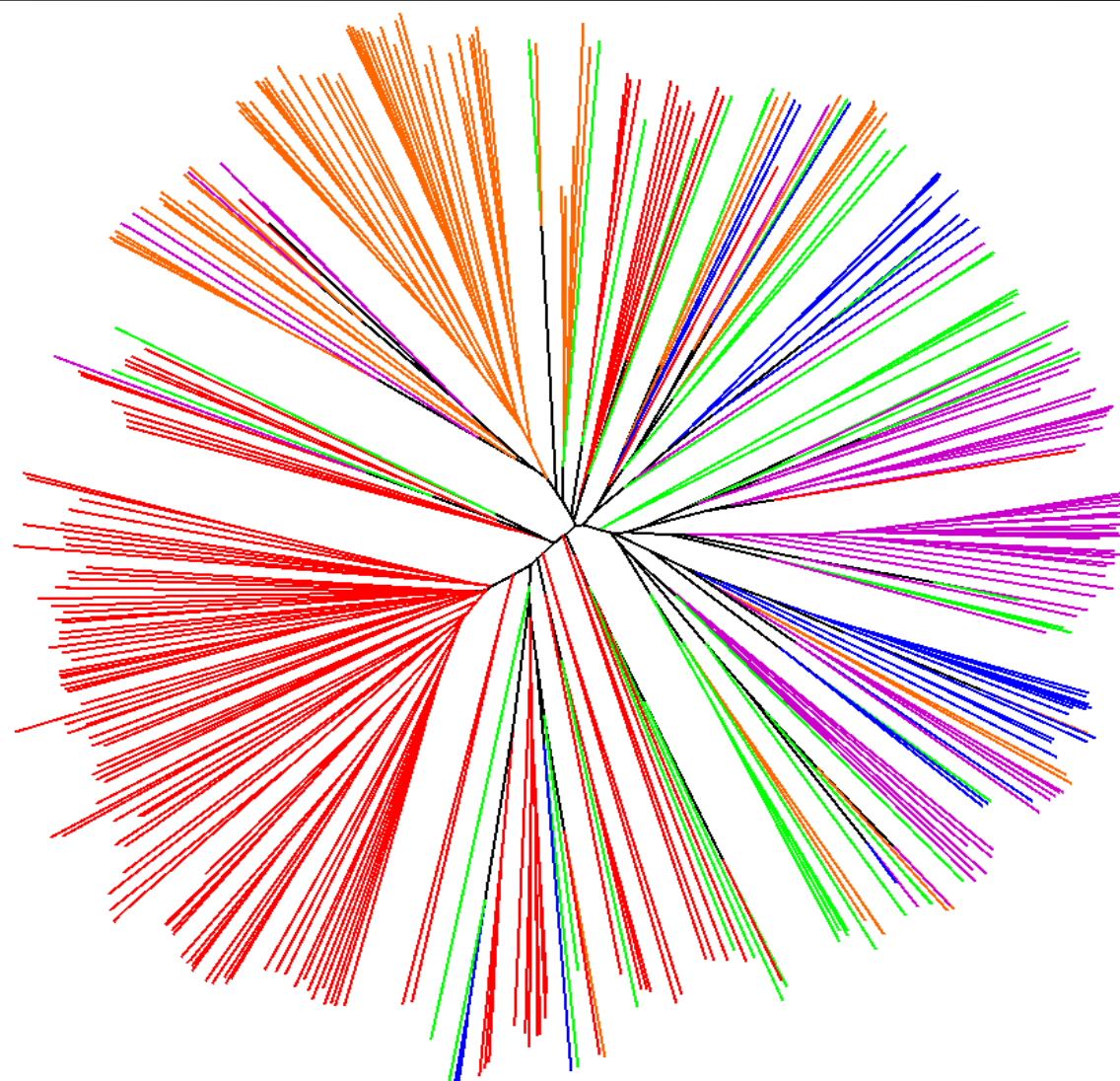

— Tropical  
— Reid  
— PA  
— PB  
— Mix

K = 4

**c**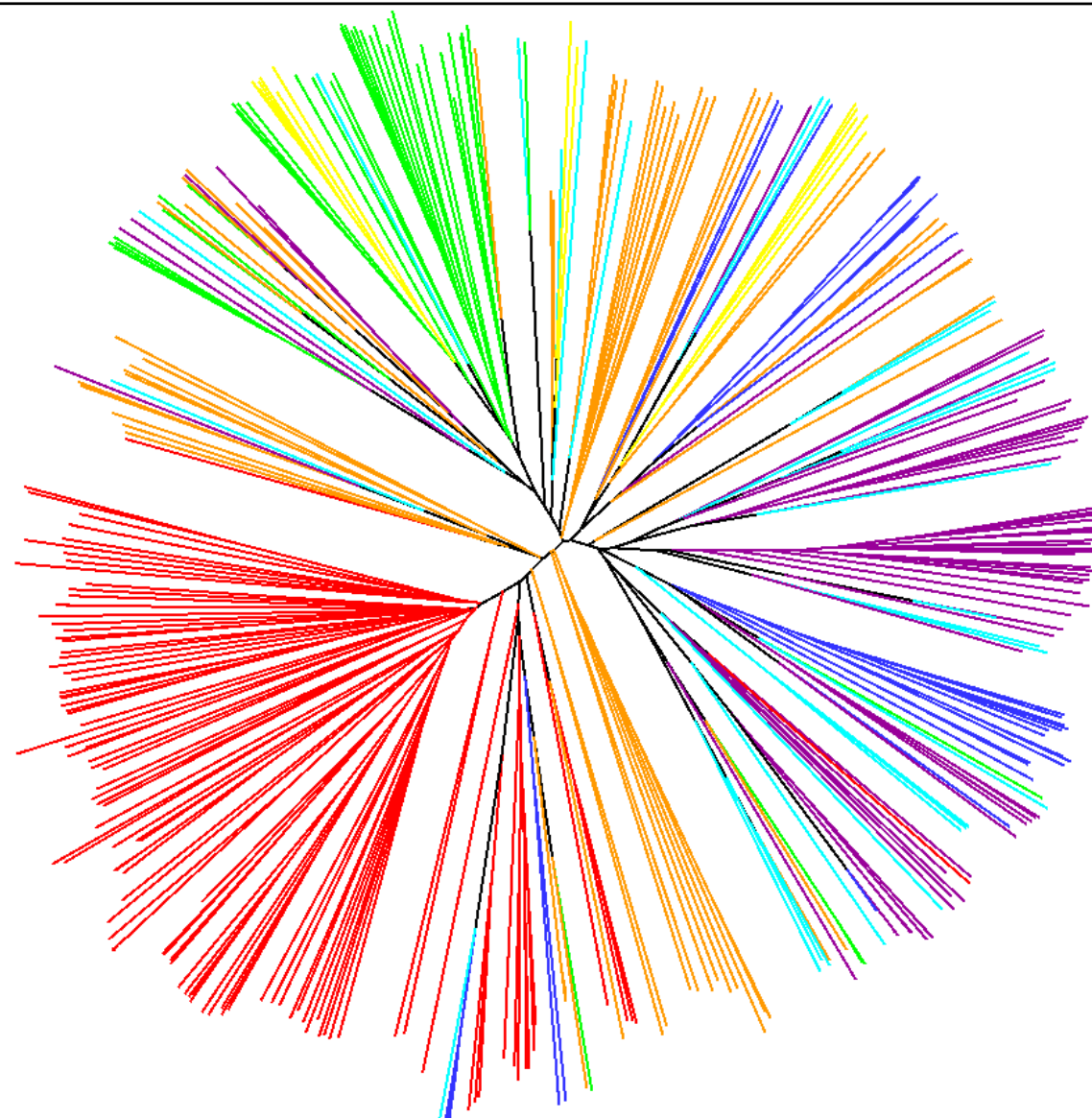

— Tropical  
— Reid  
— BSSS  
— PA  
— PB  
— North  
— Mix

K = 6

Supplement: Additional file 5: — Figure S3. Phylogenetic tree for different subgroups at K = 3, K = 4, and K = 6. (PDF 363 kb) [file 12864_2016_3041_MOESM5_ESM.pdf]

**a**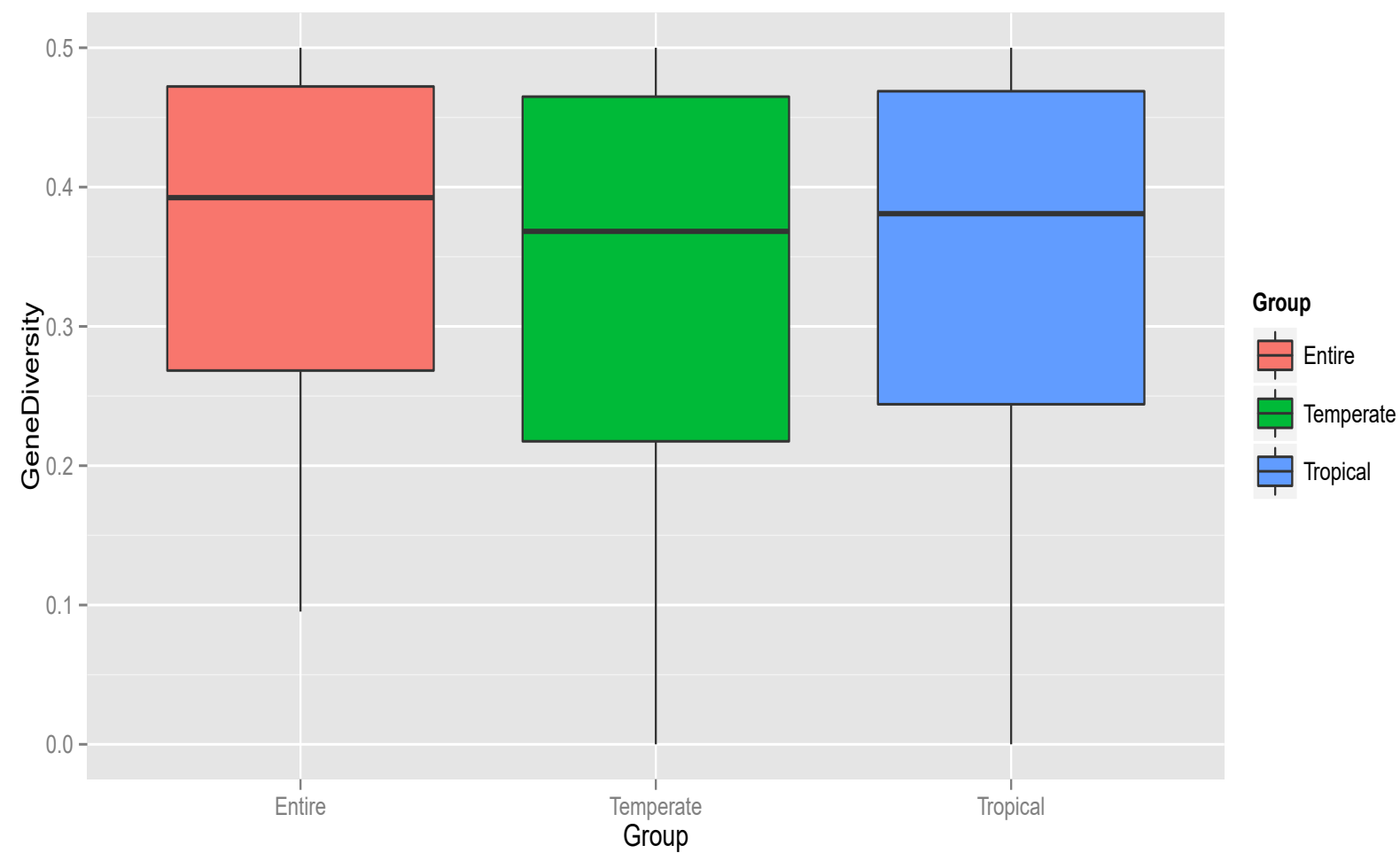**b**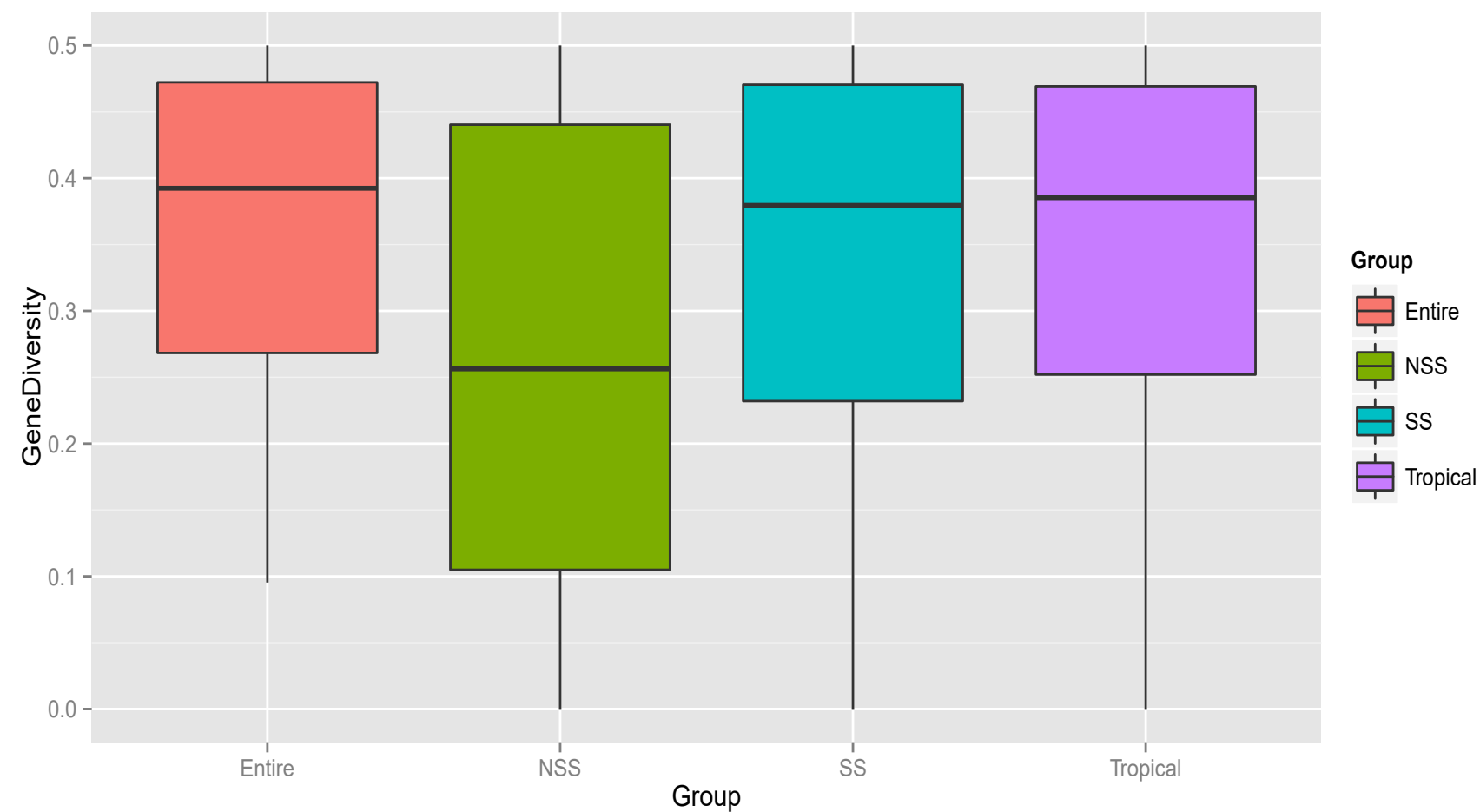**c**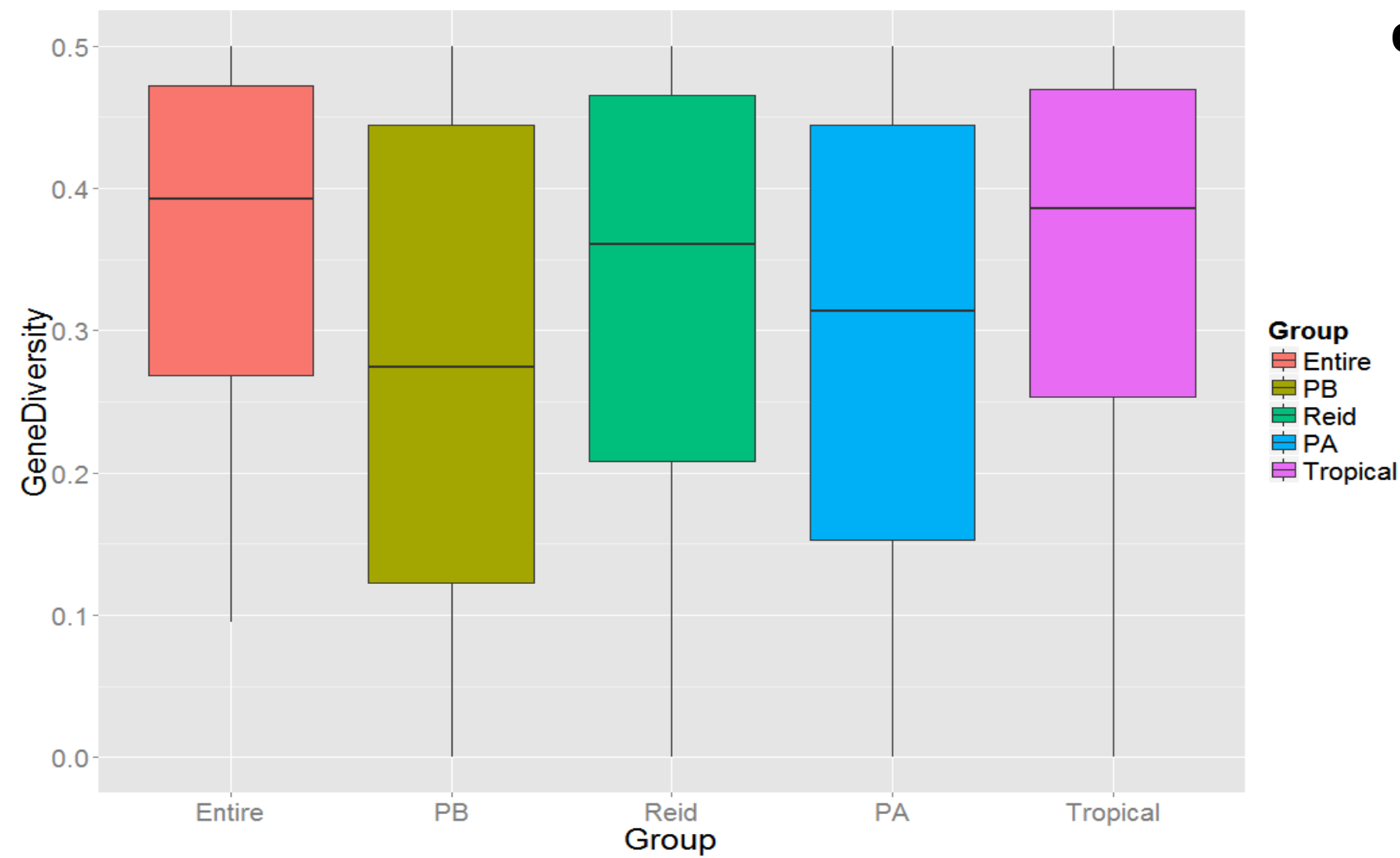**d**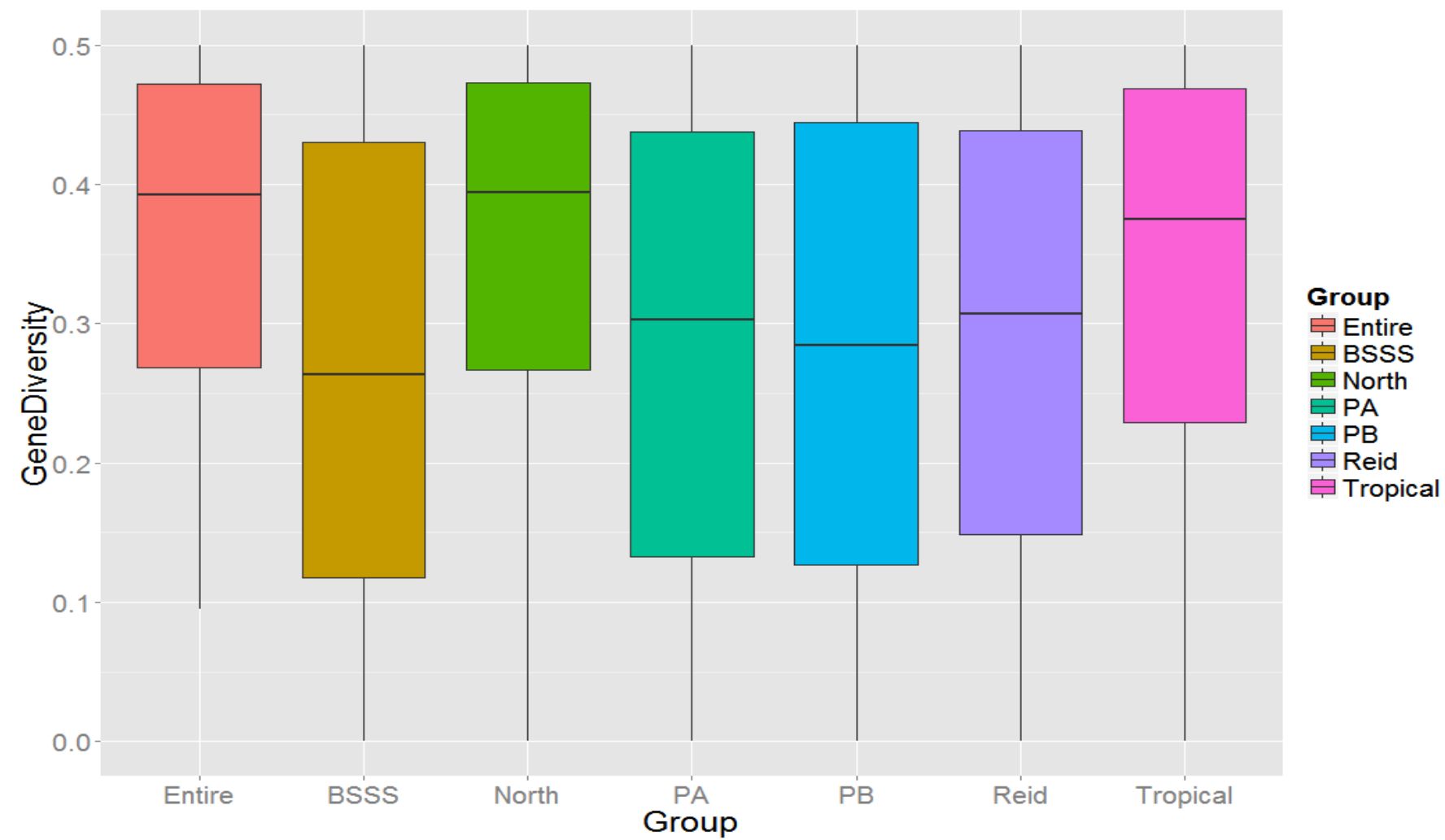

Supplement: Additional file 7: — Figure S4. Box plot of genetic diversity for each subgroup at different K values (2, 3, 4, and 6). (PDF 170 kb) [file 12864_2016_3041_MOESM7_ESM.pdf]

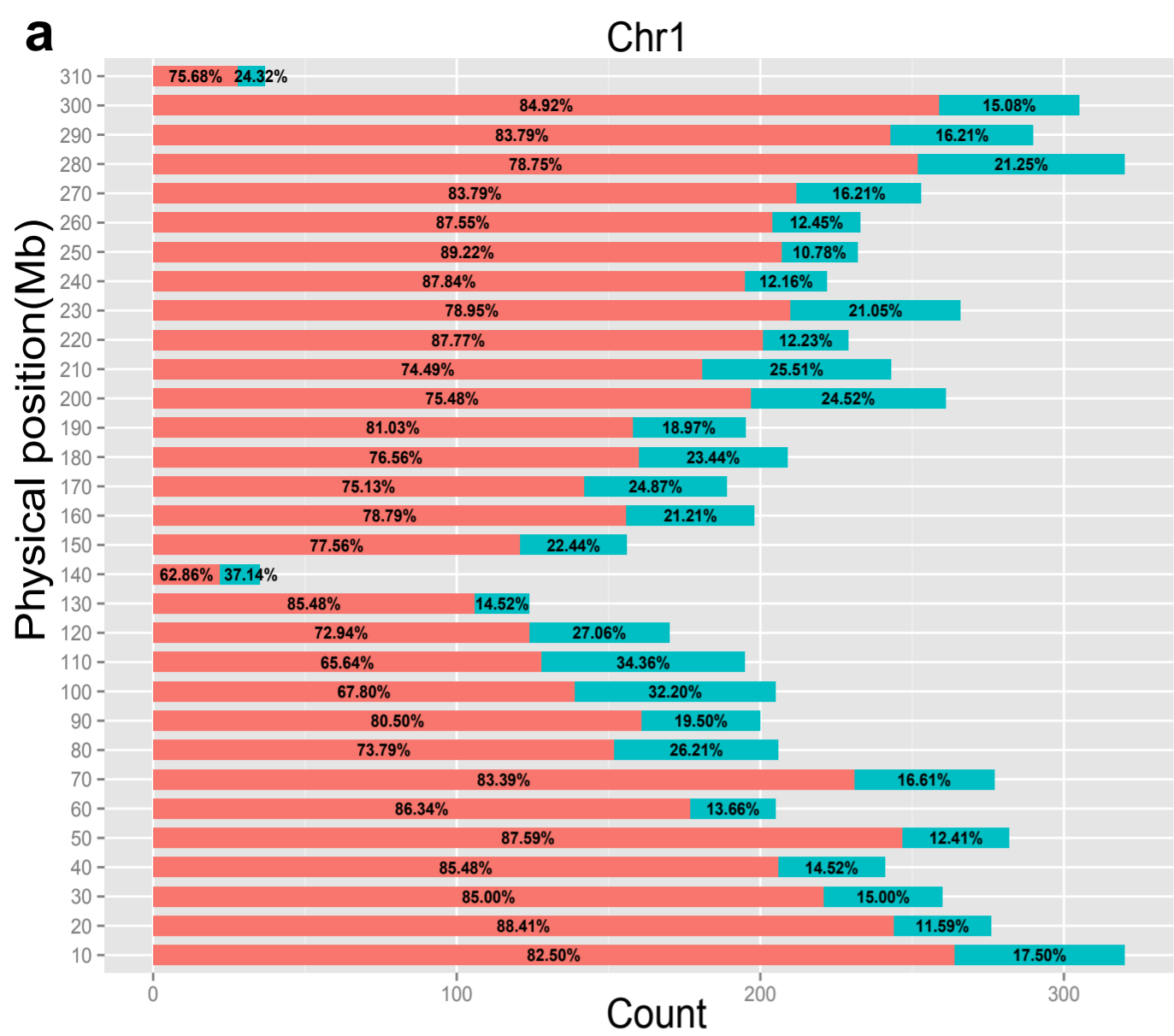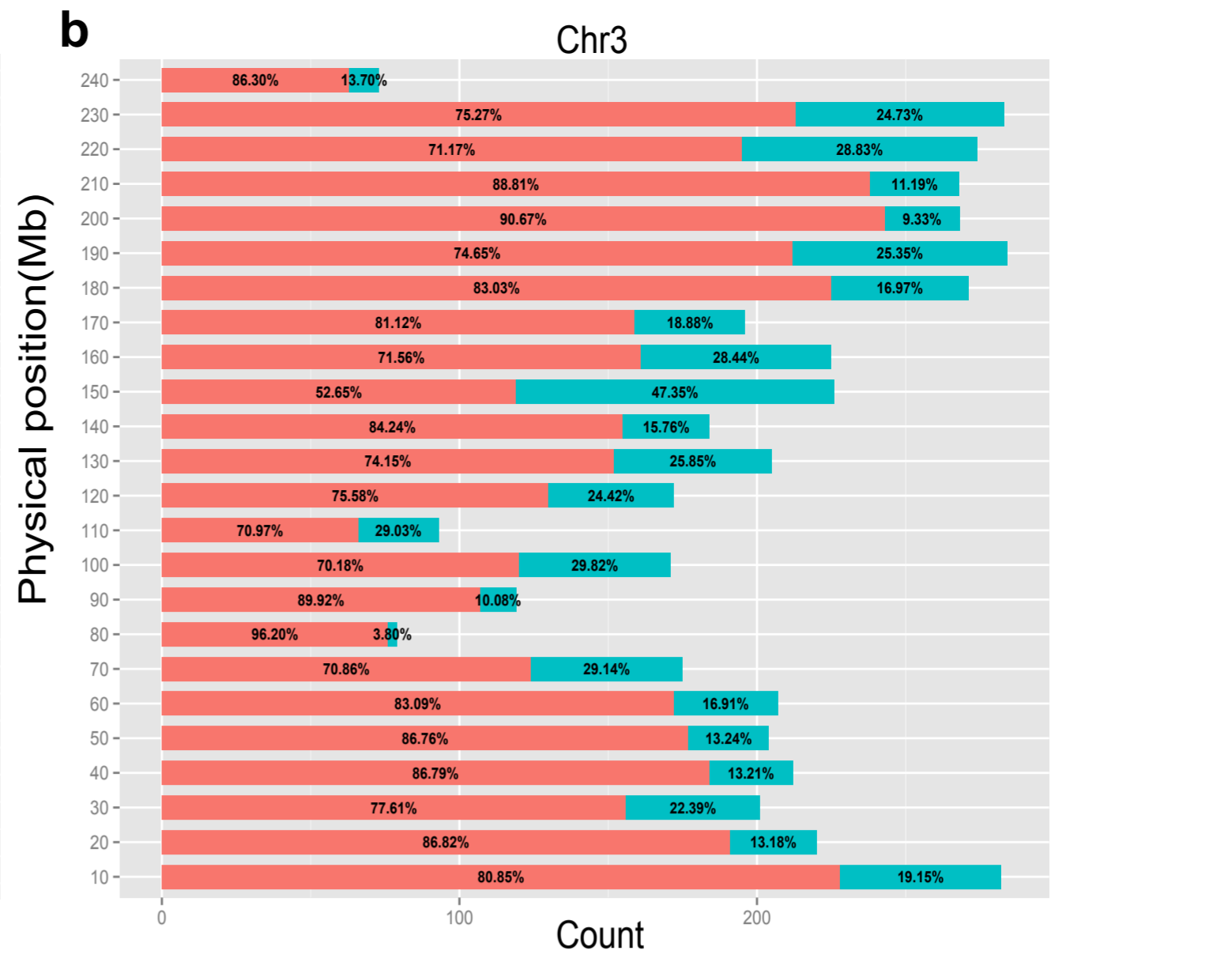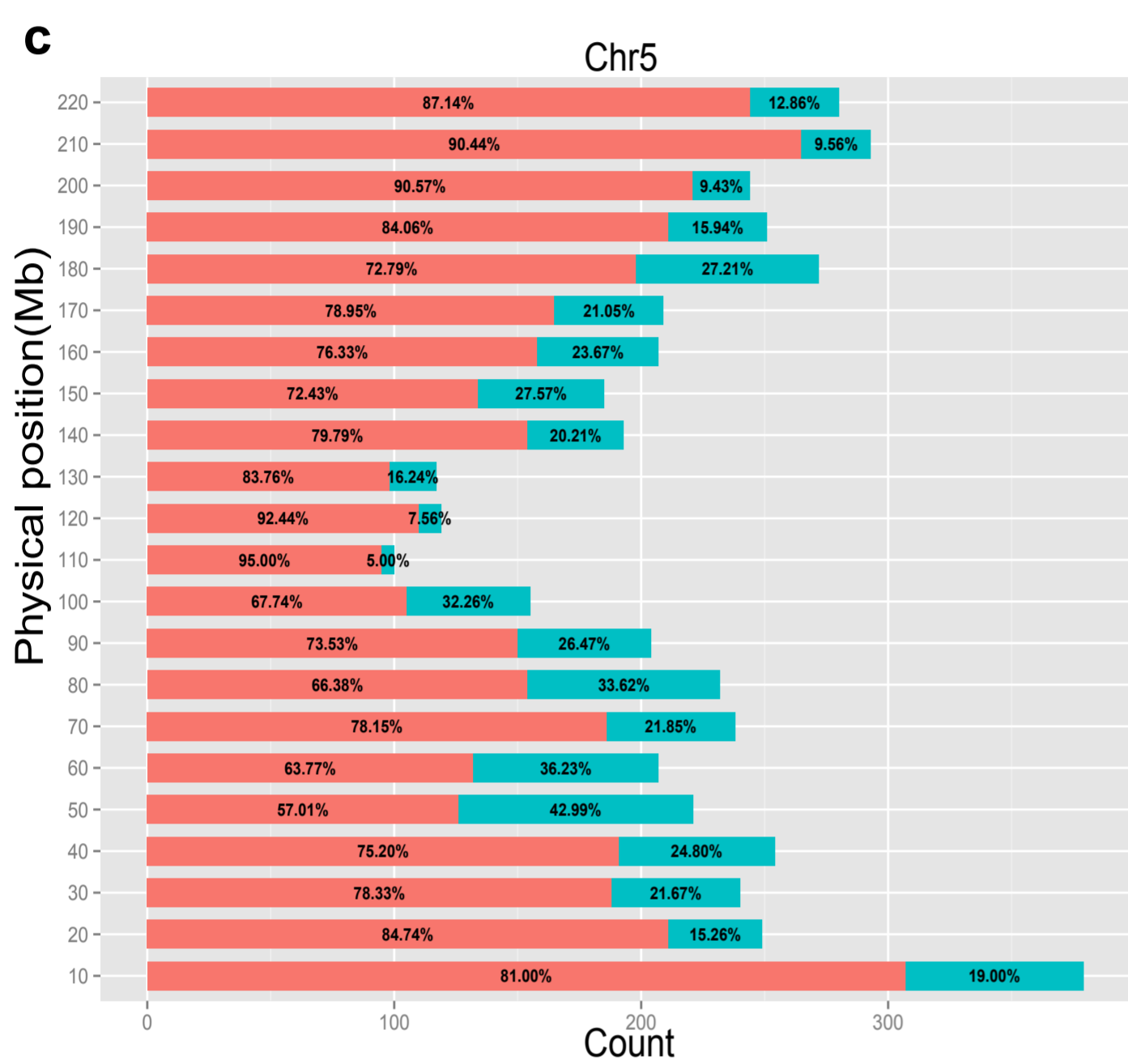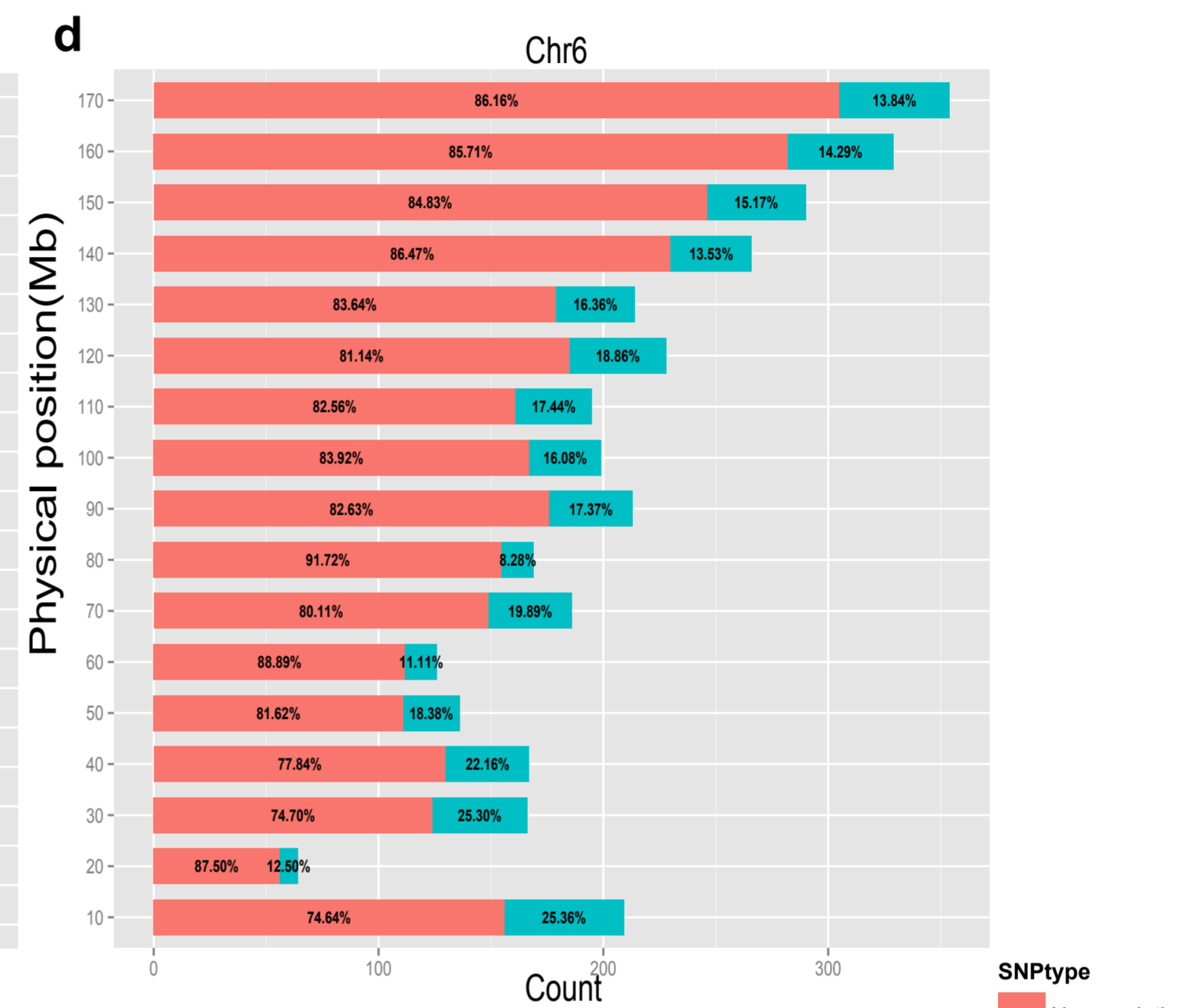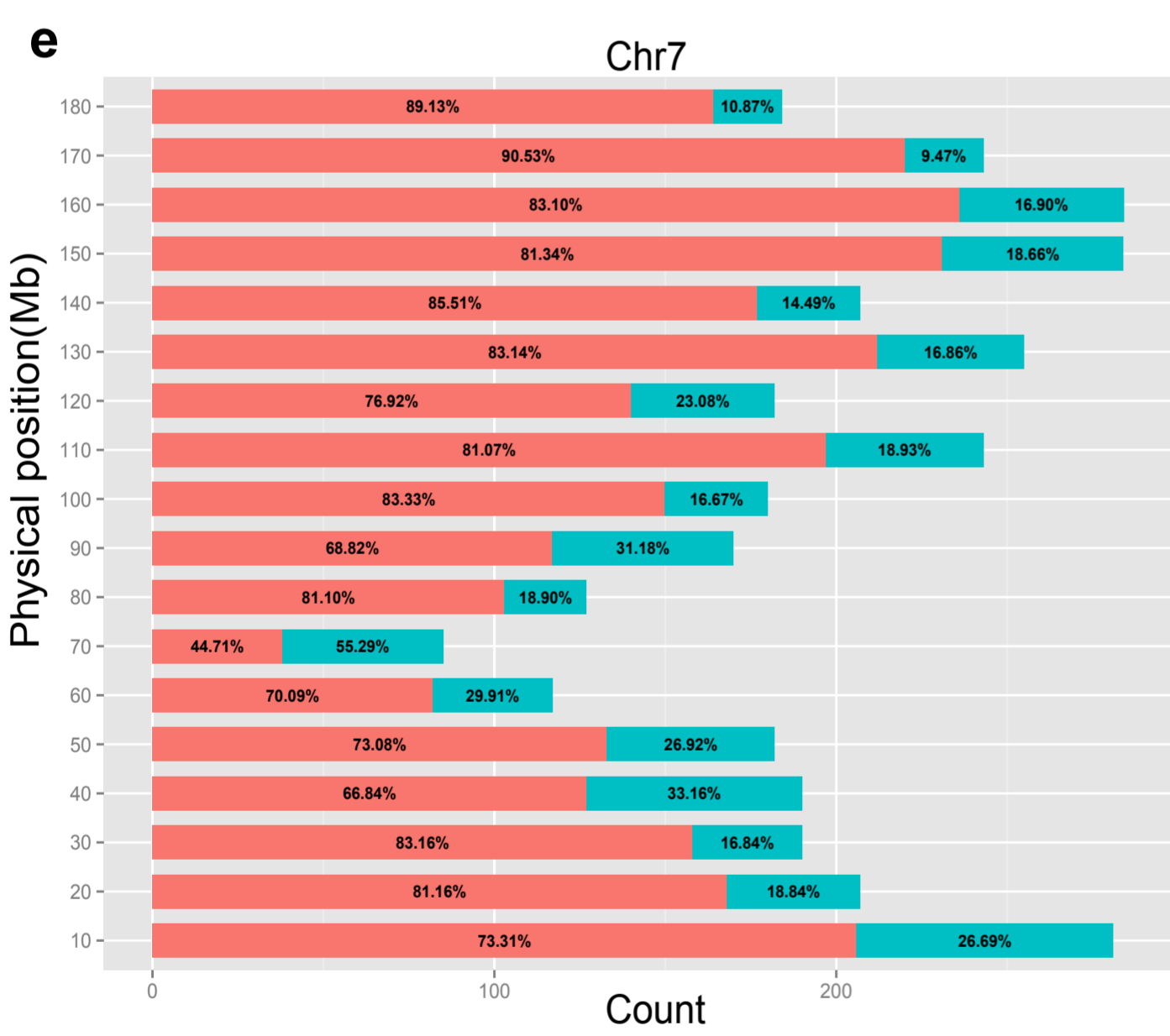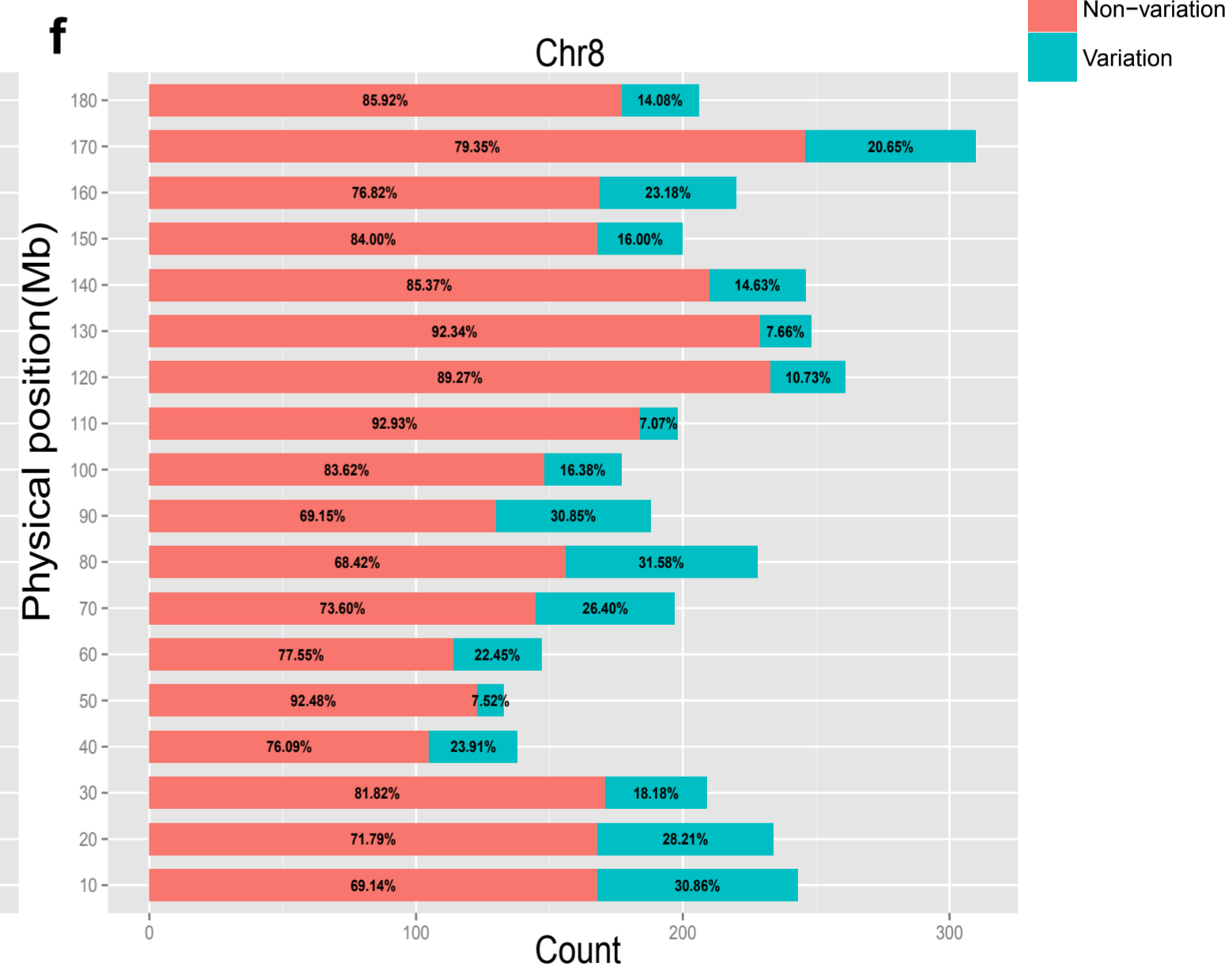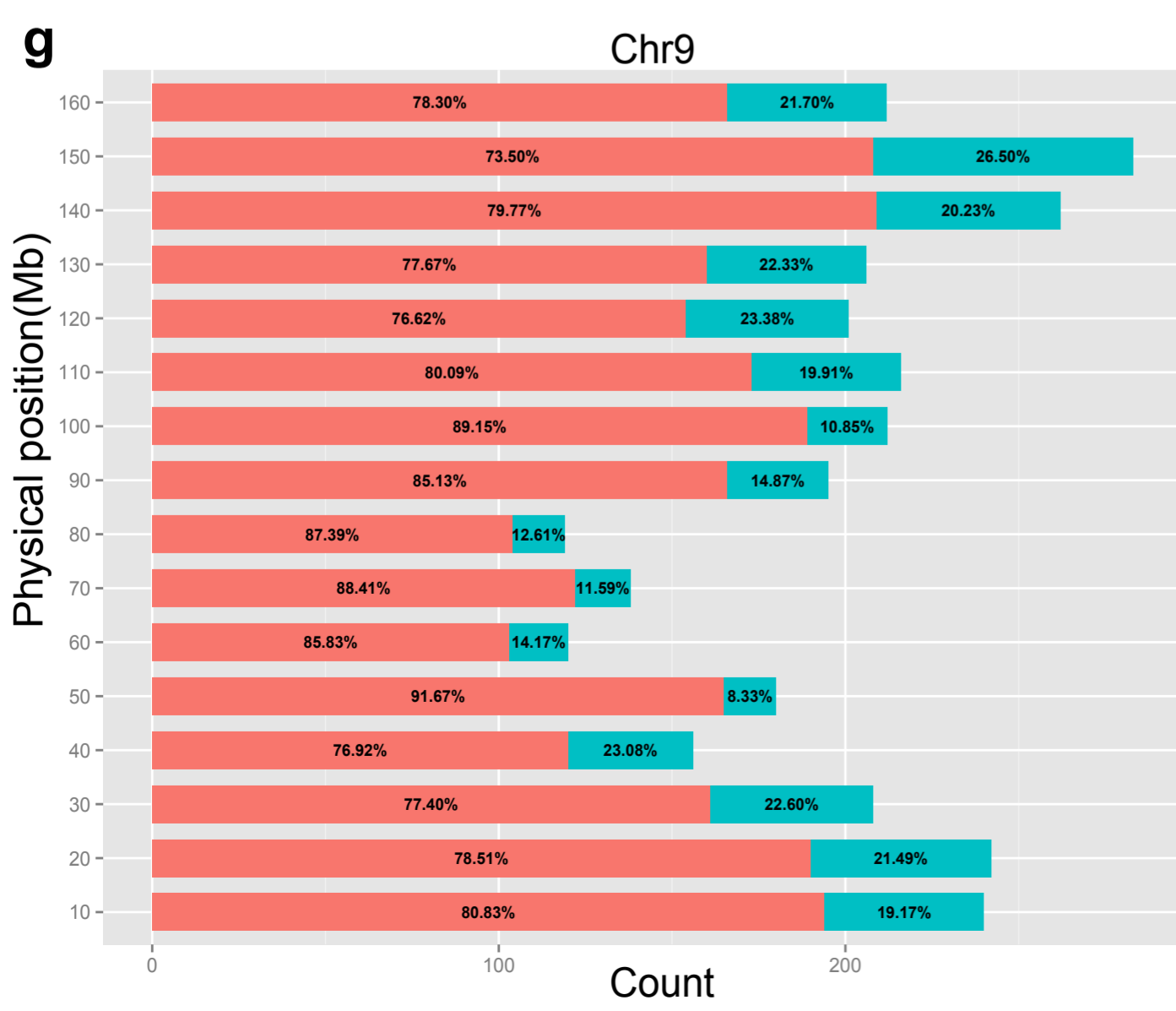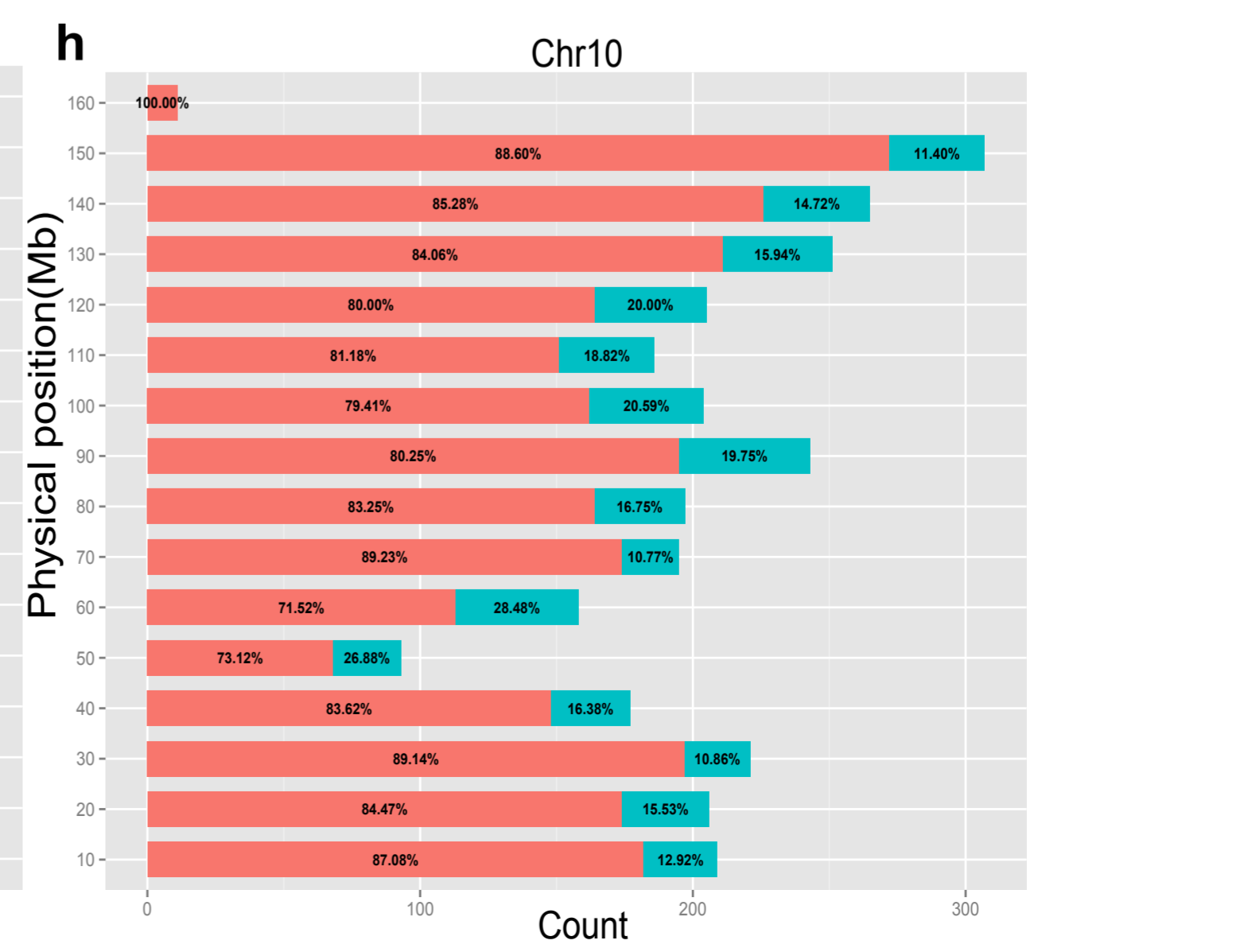

Supplement: Additional file 9: — Figure S5. Distribution of variant SNPs and the ratio of variant SNPs/total SNPs in Chr 1, 3, 5, 6, 7, 8, 9, and 10 (a–h) by 10-Mb bin size. (PDF 425 kb) [file 12864_2016_3041_MOESM9_ESM.pdf]

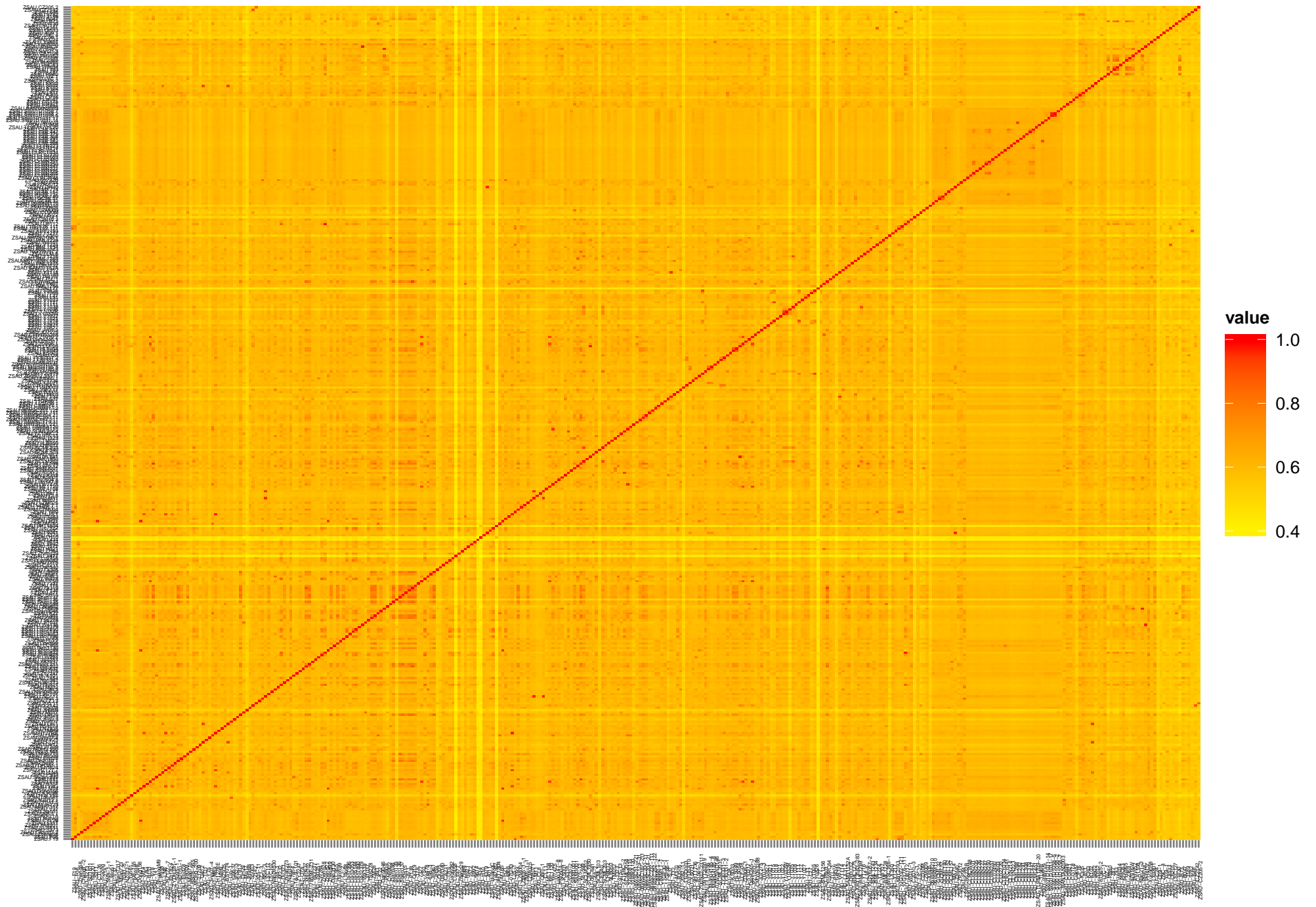

Supplement: Additional file 10: — Figure S6. Heat map of pairwise comparison between lines in the whole panel. The color gradient from yellow to red represents similarity ratio from low to high. (PDF 660 kb) [file 12864_2016_3041_MOESM10_ESM.pdf]
