# Supplementary material for: Characterizing the population structure and genetic diversity of maize breeding germplasm in Southwest China using genome-wide SNP markers
Source: BMC Genomics. 2016 Aug 31;17(1):697. doi: 10.1186/s12864-016-3041-3 (PMC5007717; doi:10.1186/s12864-016-3041-3)
Supplement: Additional file 8: — Table S4. Distribution of significant variant SNPs across 10 chromosomes (p-value < 0.001). (DOCX 13 kb) [file 12864_2016_3041_MOESM8_ESM.docx]

| Chr. | Total SNP number | Significant variant SNPs | Ratio |
| --- | --- | --- | --- |
| 1 | 6834 | 1286 | 0.188177 |
| 2 | 5177 | 1531 | 0.295731 |
| 3 | 4892 | 1026 | 0.20973 |
| 4 | 4760 | 1169 | 0.245588 |
| 5 | 4849 | 1046 | 0.215715 |
| 6 | 3511 | 587 | 0.167189 |
| 7 | 3611 | 752 | 0.208253 |
| 8 | 3783 | 739 | 0.195348 |
| 9 | 3190 | 606 | 0.189969 |
| 10 | 3128 | 516 | 0.164962 |
| Total | 43735 | 9258 | 0.211684 |
