# Supplementary material for: Characterizing the population structure and genetic diversity of maize breeding germplasm in Southwest China using genome-wide SNP markers
Source: BMC Genomics. 2016 Aug 31;17(1):697. doi: 10.1186/s12864-016-3041-3 (PMC5007717; doi:10.1186/s12864-016-3041-3)
Supplement: Additional file 11: — Table S5. Ten pairs of inbred lines with the highest pairwise similarity ratios among the entire panel. (DOCX 14 kb) [file 12864_2016_3041_MOESM11_ESM.docx]

| Pairwise inbred lines | Number of SNPs with same alleles | Ratio of number of SNPs with same alleles to total SNPs |
| --- | --- | --- |
|  |  |  |
| Zheng22 vs U8112 | 43790 | 0.992880464 |
| 08-641 vs LLF-08 | 43592 | 0.988391076 |
| H127RE vs LJS-1 | 43533 | 0.987053328 |
| Wa138 vs PH4CV | 43377 | 0.983516234 |
| PHB09 vs PH6WC | 43338 | 0.982631961 |
| M14 vs H21 | 43285 | 0.981430256 |
| SW01D1058-5 vs SW01D1058-2 | 43173 | 0.978890804 |
| 4011 vs DH40 | 43065 | 0.976442046 |
| LSC117 vs CL11 | 42975 | 0.974401415 |
| CTL26 vs H21 | 42971 | 0.97431072 |
