# Supplementary material for: Characterizing the population structure and genetic diversity of maize breeding germplasm in Southwest China using genome-wide SNP markers
Source: BMC Genomics. 2016 Aug 31;17(1):697. doi: 10.1186/s12864-016-3041-3 (PMC5007717; doi:10.1186/s12864-016-3041-3)
Supplement: Additional file 12: — Table S6. Ten pairs of inbred lines with the lowest pairwise similarity ratios among the entire panel. (DOCX 13 kb) [file 12864_2016_3041_MOESM12_ESM.docx]

| Pairwise inbred lines | Number of SNPs with same alleles | Ratio of number of SNPs with same alleles to total SNPs |
| --- | --- | --- |
| Ji477 vs 9HT1804 | 15583 | 0.353323962 |
| Ji477 vs H10 | 15806 | 0.358380192 |
| 434 vs 9HT1804 | 15856 | 0.359513876 |
| 434 vs H10 | 16057 | 0.364071286 |
| 9HT1804 vs 77 | 16233 | 0.368061854 |
| H10 vs 77 | 16248 | 0.368401959 |
| Ji477 vs FG-1 | 16416 | 0.372211137 |
| H10 vs B73 | 16435 | 0.372641937 |
| 9HT1804 vs B73 | 16466 | 0.373344821 |
| H10 vs CZ205-2 | 16831 | 0.381620715 |
