# Supplementary material for: Characterizing the population structure and genetic diversity of maize breeding germplasm in Southwest China using genome-wide SNP markers
Source: BMC Genomics. 2016 Aug 31;17(1):697. doi: 10.1186/s12864-016-3041-3 (PMC5007717; doi:10.1186/s12864-016-3041-3)
Supplement: Additional file 13: — Table S7. Similarity ratio of 30 inbred lines most similar to S37 for 10 chromosomes. (DOCX 18 kb) [file 12864_2016_3041_MOESM13_ESM.docx]

| Name | Chr1 | Chr2 | Chr3 | Chr4 | Chr5 | Chr6 | Chr7 | Chr8 | Chr9 | Chr10 | Average |
| --- | --- | --- | --- | --- | --- | --- | --- | --- | --- | --- | --- |
| A318 | 0.84 | 0.87 | 0.92 | 0.83 | 0.86 | 0.85 | 0.94 | 0.91 | 0.75 | 0.97 | 0.87 |
| BML1234 | 0.86 | 0.99 | 0.74 | 0.64 | 0.72 | 0.94 | 0.94 | 0.81 | 0.95 | 0.95 | 0.85 |
| Nan637 | 0.81 | 0.82 | 0.84 | 0.85 | 0.60 | 0.77 | 0.90 | 0.90 | 0.79 | 0.88 | 0.82 |
| Y1015 | 0.63 | 0.68 | 0.73 | 0.75 | 0.64 | 0.82 | 0.86 | 0.92 | 0.88 | 0.70 | 0.76 |
| YA3237 | 0.62 | 0.62 | 0.85 | 0.85 | 0.81 | 0.82 | 0.86 | 0.84 | 0.59 | 0.96 | 0.78 |
| 48-2 | 0.84 | 0.83 | 0.72 | 0.67 | 0.61 | 0.86 | 0.65 | 0.79 | 0.80 | 0.95 | 0.77 |
| Y1005 | 0.63 | 0.71 | 0.68 | 0.82 | 0.70 | 0.75 | 0.71 | 0.81 | 0.73 | 0.70 | 0.72 |
| MX714 | 0.67 | 0.62 | 0.85 | 0.82 | 0.60 | 0.85 | 0.87 | 0.75 | 0.68 | 0.93 | 0.76 |
| 9HT1736 | 0.70 | 0.83 | 0.76 | 0.67 | 0.63 | 0.72 | 0.83 | 0.75 | 0.73 | 0.90 | 0.75 |
| Y1114 | 0.72 | 0.74 | 0.72 | 0.74 | 0.73 | 0.68 | 0.86 | 0.71 | 0.72 | 0.70 | 0.73 |
| T32 | 0.72 | 0.73 | 0.72 | 0.74 | 0.72 | 0.67 | 0.86 | 0.71 | 0.74 | 0.71 | 0.73 |
| CIMMYT-2 | 0.74 | 0.72 | 0.73 | 0.71 | 0.68 | 0.76 | 0.83 | 0.73 | 0.77 | 0.66 | 0.73 |
| 11GD003 | 0.65 | 0.63 | 0.62 | 0.58 | 0.53 | 0.50 | 0.71 | 0.58 | 0.79 | 0.82 | 0.64 |
| 06WAM110 | 0.68 | 0.70 | 0.67 | 0.72 | 0.75 | 0.67 | 0.83 | 0.72 | 0.72 | 0.79 | 0.72 |
| F06 | 0.71 | 0.73 | 0.67 | 0.67 | 0.66 | 0.71 | 0.85 | 0.73 | 0.76 | 0.67 | 0.72 |
| 5311 | 0.69 | 0.75 | 0.73 | 0.70 | 0.71 | 0.72 | 0.79 | 0.71 | 0.70 | 0.64 | 0.71 |
| CIMMYT-1 | 0.72 | 0.74 | 0.71 | 0.65 | 0.69 | 0.63 | 0.85 | 0.71 | 0.69 | 0.72 | 0.71 |
| SN8-1-1 | 0.69 | 0.68 | 0.69 | 0.69 | 0.75 | 0.69 | 0.79 | 0.68 | 0.69 | 0.70 | 0.71 |
| GCML157 | 0.67 | 0.74 | 0.70 | 0.70 | 0.72 | 0.64 | 0.83 | 0.70 | 0.66 | 0.68 | 0.70 |
| LZM05-1-1 | 0.72 | 0.71 | 0.62 | 0.65 | 0.63 | 0.69 | 0.84 | 0.67 | 0.75 | 0.65 | 0.69 |
| S273 | 0.71 | 0.66 | 0.67 | 0.67 | 0.69 | 0.67 | 0.69 | 0.77 | 0.75 | 0.69 | 0.70 |
| FG-1 | 0.59 | 0.64 | 0.59 | 0.69 | 0.56 | 0.57 | 0.64 | 0.52 | 0.59 | 0.61 | 0.60 |
| S5003 | 0.67 | 0.69 | 0.68 | 0.67 | 0.69 | 0.56 | 0.83 | 0.67 | 0.66 | 0.70 | 0.68 |
| TLL-1 | 0.67 | 0.68 | 0.68 | 0.66 | 0.71 | 0.68 | 0.79 | 0.67 | 0.68 | 0.67 | 0.69 |
| SU1611 | 0.69 | 0.70 | 0.67 | 0.68 | 0.76 | 0.68 | 0.63 | 0.76 | 0.70 | 0.64 | 0.69 |
| ZY2247 | 0.70 | 0.67 | 0.70 | 0.64 | 0.70 | 0.64 | 0.64 | 0.64 | 0.77 | 0.73 | 0.68 |
| SW01D1031-14 | 0.65 | 0.72 | 0.70 | 0.66 | 0.67 | 0.66 | 0.83 | 0.71 | 0.66 | 0.67 | 0.69 |
| SW01D1058-7 | 0.70 | 0.67 | 0.68 | 0.78 | 0.68 | 0.67 | 0.66 | 0.68 | 0.65 | 0.72 | 0.69 |
| 10GY76-111 | 0.65 | 0.69 | 0.67 | 0.65 | 0.63 | 0.65 | 0.85 | 0.71 | 0.76 | 0.63 | 0.69 |
| LZM6-1-1 | 0.68 | 0.67 | 0.66 | 0.65 | 0.63 | 0.66 | 0.79 | 0.63 | 0.67 | 0.65 | 0.67 |
| Average | 0.70 | 0.72 | 0.71 | 0.71 | 0.68 | 0.71 | 0.80 | 0.73 | 0.73 | 0.75 |  |
