# Supplementary material for: Characterizing the population structure and genetic diversity of maize breeding germplasm in Southwest China using genome-wide SNP markers
Source: BMC Genomics. 2016 Aug 31;17(1):697. doi: 10.1186/s12864-016-3041-3 (PMC5007717; doi:10.1186/s12864-016-3041-3)
Supplement: Additional file 15: — Table S9. Ten inbred line pairs with the highest kinship coefficient among the entire panel. (DOCX 13 kb) [file 12864_2016_3041_MOESM15_ESM.docx]

| Pairwise inbred lines | Kinship value (VanRaden algorithm) |
| --- | --- |
|  |  |
| PHB09 vs PH6WC | 2.084909488 |
| Zheng22 vs U8112 | 2.063245837 |
| Wa138 vs PH4CV | 2.044662463 |
| 4011 vs DH40 | 2.037943763 |
| LSC117 vs CL11 | 1.990321091 |
| LSC117 vs M11 | 1.979039094 |
| 08-641 vs LLF-08 | 1.975160017 |
| M11vs CL11 | 1.966263614 |
| SW01D1058-5 vs SW01D1058-2 | 1.959811465 |
| M14 vs H21 | 1.951917121 |
